# Effectiveness of Conditioned Open-label Placebo With Methadone in Treatment of Opioid Use Disorder: A Randomized Clinical Trial

**DOI:** 10.1001/jamanetworkopen.2023.7099

**Published:** 2023-04-12

**Authors:** Annabelle M. Belcher, Thomas O. Cole, Ebonie Massey, Amy S. Billing, Michael Wagner, William Wooten, David H. Epstein, Stephen W. Hoag, Emerson M. Wickwire, Aaron D. Greenblatt, Luana Colloca, John Rotrosen, Lawrence Magder, Eric Weintraub, Eric D. Wish, Ted J. Kaptchuk

**Affiliations:** 1 Department of Psychiatry, University of Maryland School of Medicine, Baltimore; 2 Center for Substance Abuse Research, University of Maryland, College Park; 3 Department of Epidemiology and Public Health, University of Maryland School of Medicine, Baltimore; 4 Real-World Assessment, Prediction, and Treatment Unit, National Institute on Drug Abuse Intramural Research Program, Baltimore, Maryland; 5 Applied Pharmaceutics Lab, University of Maryland School of Pharmacy, Baltimore; 6 Sleep Disorders Center, Division of Pulmonary and Critical Care Medicine, University of Maryland School of Medicine, Baltimore; 7 Pain and Translational Symptom Science, University of Maryland School of Nursing, Baltimore; 8 Department of Psychiatry, NYU Grossman School of Medicine, New York, New York; 9 Program in Placebo Studies, Beth Israel Deaconess Medical Center, Harvard Medical School, Boston, Massachusetts

## Abstract

**Importance:**

Methadone treatment is the most effective evidence-based treatment for opioid use disorder (OUD), but challenges related to dosing and premature treatment dropout argue for adjunct interventions to improve outcomes. One potential behavioral intervention with low risk involves harnessing placebo effects.

**Objective:**

To determine the effect of a pharmacologically conditioned open-label placebo (C-OLP) on 90-day methadone dose, retention, drug use, withdrawal, craving, quality of life, and sleep.

**Design, Setting, and Participants:**

This 2-arm, open-label, single-blind randomized clinical trial was conducted between December 5, 2017, and August 2, 2019, in an academically affiliated community opioid treatment program. Analyses were conducted between October 1, 2019, and April 30, 2020. A total of 320 newly enrolled adults seeking treatment for moderate to severe OUD were assessed for study eligibility; 131 met eligibility criteria, provided informed consent, and were randomized to either C-OLP or treatment as usual (TAU) in an unequal-block (3:2) manner. Exclusion criteria were pregnancy, hospital/program transfers, and court-ordered treatment.

**Interventions:**

Participants randomized to C-OLP received pharmacologic conditioning and a placebo pill and methadone, and participants randomized to TAU were given methadone only. Participants met with the study team 5 times: at baseline (treatment intake) and 2, 4, 8, and 12 weeks postbaseline. Interactions were balanced between the 2 groups.

**Main Outcomes and Measures:**

Outcomes included 90-day methadone dose (primary) and treatment retention, drug use, withdrawal, craving, quality of life, and sleep quality (secondary). Analyses were conducted as intention-to-treat.

**Results:**

Of the 131 people enrolled in the study, 54 were randomized to TAU and 77 to C-OLP. Mean (SD) age was 45.9 (11.2) years; most of the participants were Black or African American (83 [63.4%]) and male (84 [64.1%]). No significant group differences were observed in the mean (SD) 90-day methadone dose (83.1 [25.1] mg for group TAU, 79.4 [19.6] mg for group C-OLP; *t* = 0.6219_91_; *P* = .43), but the groups differed significantly in their retention rates: 33 (61.1%) for TAU and 60 (77.9%) for C-OLP (χ^2^_1_ = 4.356; *P* = .04; number needed to treat for the beneficial outcome of 3-month treatment retention, 6; 95% CI, 4-119). C-OLP participants also reported significantly better sleep quality.

**Conclusions and Relevance:**

In this randomized clinical trial, C-OLP had no effect on the primary outcome of 90-day methadone dose. However, C-OLP participants were significantly more likely to remain in treatment. These findings support the use of C-OLP as a methadone treatment adjunct, but larger trials are needed to further examine the use of C-OLP.

**Trial Registration:**

ClinicalTrials.gov Identifier: NCT02941809

## Key Points

**Question:**

Can conditioned open-label placebo (C-OLP) help increase the efficacy of treatment with methadone for opioid use disorder?

**Findings:**

In this 2-group, single-blind randomized clinical trial including 131 individuals, 90-day retention in treatment and quality of sleep were significantly improved with C-OLP vs treatment as usual. Methadone dose, the prespecified primary outcome, did not differ significantly between groups.

**Meaning:**

The findings of this trial suggest that C-OLP may improve opioid use disorder treatment outcomes; further exploration of C-OLP as an inexpensive, low-risk adjunct to methadone treatment may be beneficial.

## Teaser

This randomized clinical trial examines the use of a pharmacologically conditioned open-label placebo in treatment of opioid use disorder.

## Introduction

First-line treatments for opioid use disorder (OUD) include medications,^1^ with one of these—methadone—possessing the largest evidence base for decreased drug use and crime and increased health improvement.^2,3,4^ But given the high-dose adverse effects (eg, constipation, nausea, or more seriously, cardiac arrhythmia),^5^ strategies rendering methadone effective at lower dosages are needed.

One possible intervention involves the harnessing of placebo effects. Broadly defined, placebo effects are improvements in symptoms attributable to the therapeutic encounter^6^ and thought to be mediated principally by expectation and conditioning^7^ and emergent theories, such as bayesian brain.^8^ Although there has been great appreciation for the potential benefits of placebos, enthusiasm has been dampened by the perception that deception is required,^8^ which would violate ethical norms of autonomy, respect, and informed consent. Recent randomized clinical trials (RCTs), mainly in patients with primary pain and other disorders,^9^ have shown, however, that deception may be unnecessary for clinically meaningful placebo effects.^10,11^ In these open-label placebo (OLP) studies, placebos are clearly identified as such and have been shown to improve outcomes for irritable bowel syndrome,^12,13,14^ chronic pain,^15,16,17,18^ allergic rhinitis,^19,20^ migraine,^21^ cancer-related fatigue,^22,23^ and menopausal hot flushes.^24^ Patients are not told the placebo will work—rather, they are informed of the possible benefits of OLPs, using data from RCTs. Frequently, these studies implement a 4-point script to provide a description, putative mechanisms, and automatic nature of OLP, and to underscore the importance of placebo adherence.^25^

An additional method of harnessing placebo effects implements pharmacologic (pavlovian) conditioning, wherein a medication’s therapeutic effects are conferred to placebos following repeated pairings with the drug.^26,27,28,29,30,31,32^ This approach has been used in studies with a primary aim of medication reduction^33,34,35,36,37^ and has been shown to treat symptoms of psoriasis,^38^ insomnia,^39^ allergic rhinitis,^40^ attention-deficit/hyperactivity disorder,^33,34^ immune suppression after kidney transplant,^41^ and postsurgical pain.^37^ To our knowledge, no studies have been conducted on either paradigm (OLP or pharmacologically conditioned placebos) or their combination for OUD. The goal of the present study was to evaluate the effect of a pharmacologically conditioned OLP (C-OLP) pill on 90-day methadone treatment outcomes, including the dose of methadone (primary) and methadone treatment retention, drug use, withdrawal, craving, quality of life, and quality of sleep (secondary). We hypothesized C-OLP would obviate the need for methadone dose escalations—an effect that would translate to a lower mean 90-day methadone dose.

## Methods

This single-site RCT was conducted between December 5, 2017, and August 2, 2019. Written informed consent was obtained from all participants. The trial protocol (Supplement 1) was approved by the institutional review board at the University of Maryland, Baltimore, and a revised version was published as a peer-reviewed article.^42^ This study report adheres to the Consolidated Standards of Reporting Trials (CONSORT) 2010 reporting guideline.^43^

### Setting and Participants

Adults seeking treatment for OUD were recruited from an urban, community-based, academically affiliated opioid treatment program. All participants met *Diagnostic and Statistical Manual of Mental Disorders, Fifth Edition*,^44^ criteria for moderate to severe OUD and were new (same-day) adult initiates for methadone treatment. Exclusions were pregnancy, other clinic or hospital transfer, and court-ordered treatment.

### Study Design and Procedures

The 12-week study included 5 individual meetings with study staff at baseline entry into treatment, and 2, 4, 8, and 12 weeks postbaseline. Participants received $25 incentives for each meeting. New patients were recruited on their first day of methadone treatment. At the end of the initial intake procedures, a member of the study team approached interested participants to obtain informed consent and completion of in-person survey and assessment. As in previous studies,^25^ a script was used as a conversational guide to emphasize 4 points: (1) a brief description of the positive impact of placebo in RCTs; (2) the automatic nature of placebo responses, with a description appropriate for lay persons of the neurobiological and psychological (conditioning) mechanisms of associative learning; (3) the lack of a requirement of belief that the placebo would work; and (4) emphasis on the criticality of placebo consumption (Supplement 1 and eTable in Supplement 2). Participants then viewed a video of a television news piece that described scientific studies of OLP interventions to treat irritable bowel syndrome.^45^ Participant characteristic data (sex, race and ethnicity) were collected as part of the baseline drug use history and assessment survey. For demographic characterization of the samples, participants self-identified their sex (female, male, other), race (selection of ≥1 of the following categories: American Indian or Alaskan Native, Asian, Black or African American, Native Hawaiian or other Pacific Islander, White, other), and ethnicity (Hispanic or Latinx: yes or no). Participants were then randomly assigned to either C-OLP or treatment as usual (TAU), were provided their observed methadone dose and, for those randomized to the intervention, a placebo pill. All group interactions with study staff were balanced for length and content, and the same outcome assessments were provided to both groups. C-OLP participants were not provided boosters or additional information regarding the rationale of the study beyond what was presented at the baseline meeting (the same content provided to TAU participants).

### Placebo Intervention and Open-label Conditioning Procedures

The intervention consisted of 2 phases: once-daily placebo conditioning (phase I, first 2 weeks) and twice-daily placebo (phase II, week 3 up to 3 months) (Supplement 1). We chose to implement a 2-phase protocol for the following reasons: (1) previous research showing that placebo responses scale with the amount of associative training delivered^26,46^; (2) best clinical practice with methadone involves a titration protocol—low doses early in treatment that are gradually increased to maintenance levels^47^—a protocol that would theoretically diminish the creation of a positive placebo response if conditioning were restricted to only a few days of methadone induction; and (3) research showing that the first 3 months of methadone treatment represent the most vulnerable period for dropout.^48,49^

### Clinic Urine Screen

Results from clinic urine drug screens (QuickTox panel; LabCorp) were logged at baseline by study staff. Substances tested included opiates, cocaine, methamphetamine, tetrahydrocannabinol, amphetamine, phencyclidine, benzodiazepine, barbiturates, methadone, oxycodone, methylenedioxymethamphetamine, buprenorphine, and fentanyl.

### Outcomes

The primary outcome was 3-month (90th-day) milligram methadone dose. Secondary outcome measures included treatment retention, self-reported drug use, opioid withdrawal, craving, quality of life, and sleep. Outcomes were assessed in person via facilitated self-report at all 5 time points, except sleep (measured only at baseline and 1 and 3 months postbaseline).

Three-month retention was assessed as a binomial variable (in treatment at day 90, yes or no). For descriptive purposes, retention was also counted as the number of days retained in treatment, from intake to day 90. We adopted the clinic’s definition of dropout (30 continuous absent days), with the last clinic visit considered the final day in treatment.

Drug use was assessed via self-report of past 2-week use of 4 common substances: opioids (including heroin, fentanyl, nonprescribed opioids), cocaine, benzodiazepines, and alcohol; other was a fifth category. Total days used (out of 14) was recorded.

Withdrawal was assessed using the objective (range, 0-13) and subjective Opiate Withdrawal Scales. Scores range from 0 to 64, with higher scores on these scales indicating greater withdrawal symptom severity.^50^

A craving assessment adapted from previous studies^51,52^ was used to measure self-reported craving intensity, using a 0 to 100 visual analog scale. Higher values indicate greater levels of craving.

Quality of life was assessed using the Abbreviated World Health Organization Quality of Life assessment. Scores range from 0 to 100, with higher scores indicating higher quality of life).^53^

Sleep was measured using the Pittsburgh Sleep Quality Index (PSQI. Scores range from 0 to 21, with higher scores indicating worse quality of sleep.^54^

### Sample Size

Power was calculated a priori for the primary outcome of the 3-month (90-day) dose of methadone. We anticipated that dose escalations after initial titration would be recommended for approximately 70% of participants in the TAU cohort in that time frame, basing this approximation on discussions with clinic staff on the proportion of patients who receive dose increases at this opioid treatment program. Thus, 60 participants per group would yield a power of 0.80 to detect a group difference if the corresponding rate in the intervention group was 44% or lower (a maximum of 26 of 60 participants), using a Fisher exact test with a 2-tailed α level of .05. This is equivalent to an odds ratio of 3.03 or a Cohen *d* value of 0.61.

### Randomization

Randomization was performed by a blinded member of the investigation team (L.C.) not directly involved with daily study procedures. A computer-generated random number sequence with unequal block randomization (60% intervention, 40% control) was created, stratified by sex, and placed into sequentially numbered opaque envelopes (n = 30/group/sex for a total of 120 random allocations). Group allocation occurred after day 1 assessment and just before the first dose of methadone with an assignment reveal.

### Blinding

At all stages of the study, methadone dose adjustments (ie, manipulations of the primary outcome) were overseen by addiction medicine physicians blinded to treatment allocation. Physicians, nurse practitioners, and counselors were also blinded, as were data analysts. Other study team members were blinded for all of day 1.

### Statistical Analysis

Analyses were conducted between October 1, 2019, and April 30, 2020. Outcomes were analyzed in accordance with randomization assignments, following an intention-to-treat approach. Descriptive statistics with central tendencies and spread were used for continuous variables; distributions and percentages were used for categorical variables. Unpaired *t* tests were used to assess group differences in mean 90th-day methadone dose (primary outcome) and mean 90th-day methadone dose in the stable-dose subgroup (subanalysis 1).

We adopted a second, more stringent analysis (subanalysis 1) to account for the potential barrier to analyzing group differences on the 90th-day methadone dose: a missed dose on the 90th day or a temporary need for a lower dose on that day due to 2 or more consecutive missed days (in line with standard opioid treatment program dosing protocols) just before the 90th day would preclude a stabilization dose of methadone, which would have potentially hidden any group differences. This analysis included only participants who had been retained for 90 days, who missed no days within the last 14 days preceding the 90th day, and whose daily dose of methadone had been stable for the 2 weeks before day 90. The rationale was that this would allow sufficient time for any therapeutically necessary dose changes to occur. We then assessed between-group differences in mean 90-day methadone dose for stable-dose participants.

Group differences were tested with χ^2^ analysis for the secondary outcome of treatment retention (continuous, up to 90 days). To illustrate the difference between the groups with respect to timing of dropout, we constructed survival curves for the distribution of time to dropout, using the Kaplan-Meier approach. We also expressed this as the number needed to treat for the beneficial outcome of 3-month treatment retention. Mixed-effects longitudinal regression models were used for all remaining secondary outcomes, including drug use, withdrawal, craving, quality of life, and sleep (global PSQI scores). All tests were 2-tailed, with an α threshold of significance set at .05. Data were stored on REDCap,^55^ and analyses were conducted using SPSS, version 27 (IBM Corp) and SAS, version 9.4 (SAS Institute Inc).

## Results

### Study Participants

**Figure 1.**
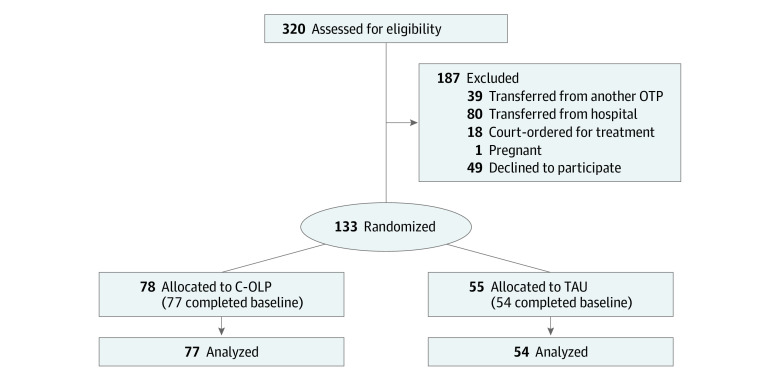
Flow Diagram of Study Participants C-OLP indicates conditioned open-label placebo; OTP, opioid treatment program; TAU, treatment as usual.

Among 320 new patients screened for methadone treatment, 131 individuals (mean [SD] age, 45.9 [11.2] years; 84 [64.1%] men; 47 [35.9%] women; 83 [63.4%] Black or African American) met eligibility criteria, provided informed consented, were randomized to receive C-OLP (n = 77) or TAU (n = 54), and completed all of day 1 and baseline procedures (Table 1 and Figure 1). Among the 187 assessed individuals who did not meet eligibility criteria, the most common reason was that they had already initiated methadone treatment (n = 119). Forty-nine people declined participation, with most due to time constraints. Seven individuals did not want to add anything to their treatment. Participants reported a mean (SD) of 22.9 (12.8) years of heroin and/or fentanyl use, and 66 (50.4%) reported current intravenous drug use. Two individuals who had provided informed consent were removed from treatment before methadone dosing following intake: one was hospitalized for acute edema and the other was referred for office-based buprenorphine treatment; these individuals’ data are not included in any reported analyses. Three individuals allocated to the C-OLP group who had completed baseline assessments withdrew from the study following 1, 3, and 8 days of intervention.

**Table 1.** Participant Baseline Characteristics

Characteristic	Patients, No. (%)		
	TAU (n = 54)	C-OLP (n = 77)	Total (N = 131)
Age, mean (SD), y	43.5 (11.2)	47.6 (10.9)	45.9 (11.2)
Sex			
Female	23 (42.6)	24 (31.2)	47 (35.9)
Male	31 (57.4)	53 (68.8)	84 (64.1)
Race			
Black or African American	31 (57.4)	52 (67.5)	83 (63.4)
White	23 (42.6)	24 (31.2)	47 (35.9)
Other^a^	0	1 (1.3)	1 (0.8)
Ethnicity (Hispanic or Latinx)	1 (2.0)	0	1 (0.8)
Past 12-mo income, $			
<15 000	47 (87.0)	67 (87.0)	114 (87.0)
15 000-19 999	5 (9.3)	6 (7.8)	11 (8.4)
20 000-39 999	2 (3.7)	1 (1.3)	3 (2.3)
>40 000	0	3 (3.9)	3 (2.3)
Past 12-mo occupation^b^			
Unemployed	29 (53.7)	39 (50.6)	68 (51.9)
Retired or disabled	13 (24.1)	19 (24.7)	32 (24.4)
Employed parttime	4 (7.4)	10 (13.0)	14 (10.7)
Employed full time	2 (3.7)	6 (7.8)	8 (6.1)
Homemaker/caregiver	5 (9.3)	3 (3.9)	8 (6.1)
Military service	1 (1.9)	0	1 (0.8)
Incarcerated	0	1 (1.3)	1 (0.8)
No. of years opioid use, mean (SD)	22.4 (12.4)	23.3 (13.2)	22.9 (12.8)
Intravenous drug use	34 (63.0)	32 (41.6)	66 (50.4)
Positive clinic urine toxicology screen at baseline^c^			
Opiates^d^	53 (98.1)	69 (89.6)	122 (93.1)
Cocaine	43 (79.6)	52 (67.5)	95 (72.5)
Methamphetamine	3 (5.6)	3 (3.9)	6 (4.6)
Tetrahydrocannabinol	11 (20.4)	19 (24.7)	30 (22.9)
Benzodiazepines	21 (38.9)	23 (29.9)	44 (33.6)

Abbreviations: C-OLP, conditioned open-label placebo; TAU, treatment as usual.

^a^ One individual self-identified as Pacific Islander and White.

^b^ Options were not mutually exclusive; 1 participant reported both unemployed and incarcerated.

^c^ Data missing from 3 participants who were unable to provide a biological sample on the date of intake. Reported percentages are inclusive of missing data.

^d^ Positive for at least 1 of the following drugs: morphine, oxycodone, fentanyl, buprenorphine, or methadone.

### Primary Outcome: 3-Month Dose of Methadone

**Figure 2.**
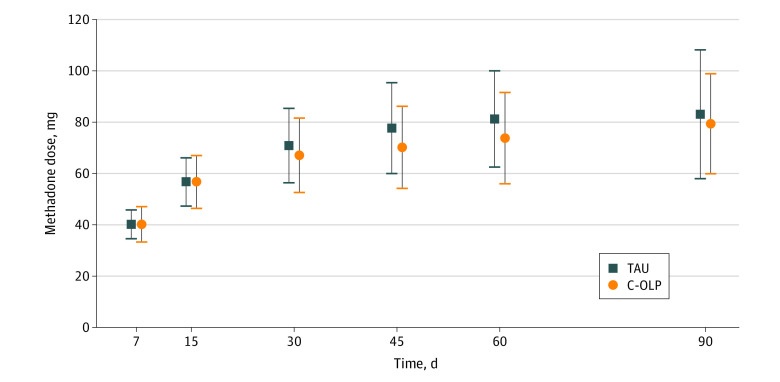
Mean Methadone Doses at Various Intervals Up to 90 Days Mean group methadone doses are shown for postentry into treatment. Mean values include data that were available for people retained at each time point. Treatment as usual (TAU): 47 individuals retained at 7 days, 43 at 15 days, 38 at 30 days, 37 at 45 days, 34 at 60 days, and 33 at 90 days. Conditioned open-label placebo (C-OLP): 73 individuals retained at 7 days, 71 at 15 days, 67 at 30 days, 65 at 45 days, 64 at 60 days, and 60 at 90 days. Whiskers indicate 95% CIs.

We report findings for all 131 participants in an intention-to-treat analysis. Fifty-four individuals were randomized to TAU, 77 to C-OLP. Starting methadone doses for both groups ranged from 10 to 40 mg (mode = 25 mg). There were no statistically significant differences between the groups in their mean (SD) dose at day 90: 83.1 (25.1) mg for group TAU (n = 33) and 79.4 (19.6) mg for group C-OLP (n = 60) (*t*_91_ = 0.6219; *P* = .43). Group mean doses were also similar across the first 90 days (Figure 2).

Subanalysis 1 included only individuals who had maintained a stable dose of methadone during the last 2 weeks of treatment (15 TAU, 32 C-OLP). There was no significant difference between the groups in mean (SD) 90th-day methadone dose (85.3 [13.4] mg for TAU, 87.0 [15.6] mg for C-OLP; t_45_ = 0.3621; *P* = .72).

### Secondary Outcomes

**Figure 3.**
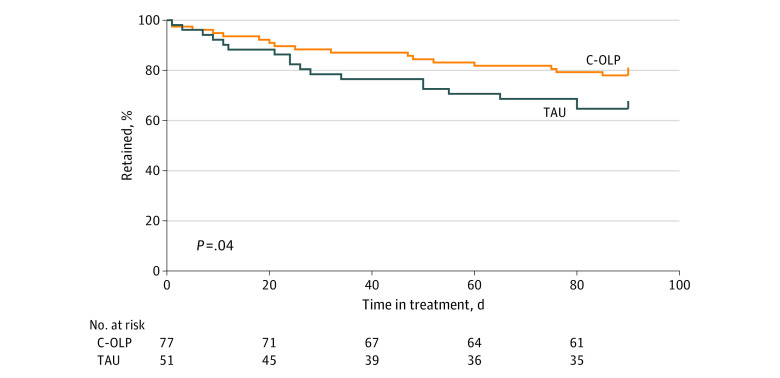
Probability of Treatment Retention by Group Estimated by the Kaplan-Meier method overall probability function. C-OLP (n = 77) overall probability, 0.75 (95% CI, 0.66-0.86); TAU (n = 54) overall probability, 0.59 (95% CI, 0.48-0.74). C-OLP indicates conditioned open-label placebo; TAU, treatment as usual.

A total of 33 of 54 TAU participants (61.1%) and 60 of 77 C-OLP participants (77.9%) remained in treatment at 90 days (χ^2^_1_ = 4.356; *P* = .04), reflecting greater retention for C-OLP participants; the number needed to treat for the beneficial outcome of 3-month treatment retention, 6 (95% CI, 4-119) (Figure 3).

Mixed-effects models showed that group C-OLP reported better sleep quality (global C-OLP PSQI score, 8.12 [0.48] vs 9.9 [0.46] for TAU; *P* = .047), an estimated mean PSQI score difference of 1.79 points. No statistically significant differences were found in any other outcome measure (eTable in Supplement 2).

## Discussion

Our primary aim was to test whether a C-OLP intervention could improve methadone treatment outcomes. Our hypothesis that 90-day methadone doses would be lower for C-OLP than for TAU recipients was not supported. We can only speculate as to why there were no significant differences between the groups, but due to its bioavailability, clearance, and half-life, methadone dosing is highly individualized.^47^ Furthermore, to avoid the risk of overdose, a patient’s methadone dose is affected by missed days, which can result in dose cuts or the need for reassessment and reinitiation. These influences may have precluded the ability to observe any potential group differences in methadone dose.

Unexpectedly, we found a significant difference in the groups’ 90-day retention rates, with 61.1% retention attrition in the TAU group and 77.9% for group C-OLP (Figure 3). The observed TAU attrition rate is very similar to the 40% attrition reported in the comparison cohort of a recently published trial conducted at the same opioid treatment program,^56^ reinforcing the reliability of our TAU retention findings.

Additionally, secondary analyses suggested another benefit in the C-OLP group: relative to the TAU participants, they reported better sleep over the first 90 days of treatment, with an estimated mean PSQI score difference of 1.78 points (Table 2). Differences greater than 3 on the PSQI are generally considered clinically meaningful; however, 2 separate systematic reviews have identified differences between 1.54 and 3 as clinically meaningful.^57,58^ Disrupted sleep can be a major challenge for patients with OUD,^59,60^ and several trials are focusing on sleep as a therapeutic target for OUD (eg, NCT04287062). Sleep itself may have large placebo effects that can be manipulated by conditioning paradigms.^39,61^ Thus, our findings are consistent with prior research and suggest that studies should incorporate more detailed examinations of sleep as a mechanism of OUD treatment-related changes.

**Table 2.** Estimated Mean of Secondary Outcomes With Mixed Effects Longitudinal Regression Estimates in the TAU (n = 54) and C-OLP (n = 77) Groups

Domain	Variable	Group	Months after baseline estimate, mean (SE)			Global *P* value
			1	2	3	
Self-reported past 2-wk drug use, d	Opiates	TAU	5.80 (0.66)	4.86 (0.74)	5.75 (0.75)	.46
		C-OLP	4.96 (0.74)	4.69 (0.55)	4.45 (0.56)	
	Cocaine	TAU	2.28 (0.70)	3.47 (0.77)	2.36 (0.78)	.62
		C-OLP	2.42 (0.57)	2.65 (0.59)	2.93 (0.60)	
	Benzodiazepines	TAU	0.57 (0.38)	0.46 (0.41)	0.73 (0.42)	.79
		C-OLP	0.19 (0.31)	0.28 (0.32)	0.31 (0.32)	
	Alcohol	TAU	0.48 (0.36)	0.61 (0.40)	0.33 (0.40)	.94
		C-OLP	0.54 (0.29)	0.87 (0.30)	0.54 (0.30)	
	Other drug use	TAU	1.54 (0.52)	2.05 (0.55)	1.58 (0.56)	.44
		C-OLP	2.17 (0.45)	1.87 (0.46)	2.12 (0.46)	
Withdrawal	Objective opiate withdrawal	TAU	1.72 (0.22)	1.76 (0.24)	1.45 (0.25)	.21
		C-OLP	1.46 (0.17)	1.21 (0.18)	1.08 (0.18)	
	Subjective opiate withdrawal	TAU	17.2 (2.3)	17.5 (2.6)	14.0 (2.6)	.19
		C-OLP	14.6 (1.9)	11.4 (1.9)	9.4 (2.0)	
Craving	Adapted craving score	TAU	9.69 (1.06)	9.36 (1.18)	8.68 (1.20)	.06
		C-OLP	6.84 (0.85)	6.61 (0.89)	6.45 (0.90)	
Quality of life: WHOQOL-BREF^a^	Physiological health score domain	TAU	53.2 (2.3)	51.5 (2.3)	54.9 (2.3)	.82
		C-OLP	52.0 (1.7)	53.1 (1.8)	54.0 (1.8)	
	Psychological health score domain	TAU	62.1 (2.3)	60.6 (2.5)	62.5 (2.6)	.99
		C-OLP	61.8 (1.9)	59.9 (2.0)	62.2 (2.0)	
	Social relationships score domain	TAU	56.1 (3.5)	62.2 (3.8)	65.4 (3.9)	.13
		C-OLP	64.8 (2.9)	65.1 (3.0)	64.1 (3.0)	
	Environment score domain	TAU	58.4 (2.6)	56.4 (2.8)	59.9 (2.2)	.89
		C-OLP	57.9 (2.2)	58.1 (2.2)	59.1 (2.3)	
Sleep	Sleep: global PSQI score^b^	TAU	9.92 (0.56)	NA	9.90 (0.46)	.047
		C-OLP	9.04 (0.46)	NA	8.12 (0.48)	

Abbreviations: C-OLP, conditioned open-label placebo; NA, not applicable; PSQI, Pittsburgh Sleep Quality Index; TAU, treatment as usual; WHOQOL-BREF, World Health Organization Quality of Life assessment.

^a^ Scores range from 0 to 100, with higher scores indicating higher quality of life.

^b^ Scores range from 0 to 21, with higher scores indicating worse quality of sleep.

Our unique implementation combined 2 methods to harness placebo effects: OLP and pharmacologic conditioning. In open-label studies, no pretense is made concerning the fact that the pill or device is physiologically inert. Patient participants are oriented transparently to the possible beneficial effects of placebos. We did not guide participant expectations regarding our specific study aims; rather, participants were informed of the nonspecific therapeutic benefits that can accompany placebo therapy but were not informed of the operationalization of assessments.

Few studies have combined conditioning and OLP. In a novel small feasibility study, Morales-Quezada and colleagues^36^ randomized inpatients with spinal cord injury to either C-OLP or TAU and compared subsequent opioid consumption rates and self-reported pain. The C-OLP group showed less opioid consumption and less pain relative to TAU. Flowers et al^37^ advanced these findings to conduct a larger RCT in patients following back surgery (again, the primary outcome was opioid consumption), and similarly found that patients receiving C-OLP consumed 30% less opioids for postoperative pain (−14.5 daily morphine milligram equivalents; 95% CI, −26.8 to −2.2 daily morphine milligram equivalents) and reported lower daily worst-pain scores (21.0 points; 95% CI, −2.0 to −0.1 points) than patients receiving TAU.^37^ These findings underscore the relevance of C-OLP in opioid paradigms.

Although our results do not speak to mechanism, the beneficial effect of C-OLP on methadone treatment retention could be related to the benefits observed in sleep quality or other not-yet-assessed effects on overall function. As part of this RCT, we also obtained qualitative data from C-OLP participants to understand perceptions of efficacy—data that could help elucidate perceived benefits of C-OLP. A forthcoming study will represent these data in full.

The clinical implications of the C-OLP intervention described in this study are great. Retention in treatment is a serious challenge for the field of addiction medicine and calls abound for an all hands on deck approach to identify solutions to stave overdose rates and increase treatment engagement and retention. We have demonstrated the general feasibility of administering a placebo adjunct to standard-of-care methadone in a community-based opioid treatment setting. Our experience running this trial showed us that C-OLP did not produce a significant burden to clinic procedures. Future studies should rigorously evaluate implementation outcomes, however (eg, patient and staff acceptability, measures of feasibility), to better inform the practicality of implementing a placebo adjunct to methadone treatment. Notwithstanding, the low-cost, low-risk nature of this intervention suggests that C-OLP could provide an appealing strategy to target early methadone treatment adherence. Well-powered studies are needed to evaluate this intervention’s efficacy on methadone treatment retention.

### Strengths and Limitations

Strengths of our study include the use of between-group structural equivalence and blind assessment. Furthermore, outcomes of interest (methadone doses and number of treatment days) were based on objective measures, extracted from patient records.

This trial has limitations. It was not powered to detect group differences for outcomes other than methadone dose; secondary outcome differences could be artifactual. Additionally, open-label interventions are inherently incompatible with double-blinding. Previous open-label RCTs have been only assessor blinded; ours is one of the few in which the blind extended to the clinicians. Personal communications between the lead author (A.M.B.) and these individuals (treatment physicians [A.D.G., E.W.] and the nurse practitioner) suggest that this blind was not broken (ie, clinicians were not even aware of their patients’ study involvement). Despite this, the possibility still exists that C-OLP participants discussed the study with treatment team members. Furthermore, this study was conducted in a single setting—findings from which may not be generalizable to other treatment settings and populations. A planned larger trial will address this shortcoming. A final limitation is that we did not quite achieve our intended sample size of 60 participants in the TAU group.

## Conclusions

To our knowledge, this is the first RCT to assess the efficacy of a harnessed placebo intervention in a clinical OUD treatment context. Our findings of C-OLP–enhanced treatment retention and ameliorated sleep merit further investigation, especially considering the unchecked OUD epidemic that continues in the US.

## Notes

Supplement 1.
Trial Protocol and Statistical Analysis Plan

Supplement 2. **eTable.** Observed (Raw) Means of Secondary Outcomes at Each Time Point: Drug Use, Withdrawal, Craving, Quality of Life, and Sleep (TAU, n = 54; C-OLP, n = 77)

Supplement 3.
Data Sharing Statement
