# Supplementary material for: Effectiveness of Conditioned Open-label Placebo With Methadone in Treatment of Opioid Use Disorder: A Randomized Clinical Trial
Source: JAMA Netw Open. 2023 Apr 12;6(4):e237099. doi: 10.1001/jamanetworkopen.2023.7099 (PMC10099063; doi:10.1001/jamanetworkopen.2023.7099)
Supplement: Supplement 1. — Trial Protocol and Statistical Analysis Plan [file jamanetwopen-e237099-s001.pdf]

PROTOCOL

Conditioned Open-Label Placebo for Methadone Treatment of Opioid Use Disorder: A Single-Blind Randomized Clinical Trial

Principal Investigator: Annabelle M. Belcher, PhD; abelcher@som.umaryland.edu

HRPO Record: HP-00070829

Date: July 19, 2017

Sponsor: The Foundation for the Science of the Therapeutic Encounter (F-STE)

Study Site:
University of Maryland Addiction Treatment Center (UMATC)
1001 W. Pratt Street
Baltimore, MD 21223
443-462-3400

IRB of Record:
University of Maryland, Baltimore, Professional Schools IRB
Human Research Protections Office
620 W. Lexington Street, 2nd Floor
Baltimore, MD, 21201
410-706-5037
hrpo@umaryland.edu

INDEX

1.0 BACKGROUND

2.0 PROJECT OVERVIEW
- 2.1 Brief project overview
- 2.2 Specific Aims
- 2.3 Significance

3.0 FACILITIES AND RESOURCES
- 3.1 University of Maryland Addiction Treatment Center (UMATC)
- 3.2 University of Maryland School of Pharmacy Good Manufacturing Practice Facility

4.0 CLINICAL OUD DIAGNOSIS AND TREATMENT

5.0 RESEARCH DESIGN AND METHODS
- 5.1 Overview of study design
- 5.2 Study population
- 5.3 Eligibility criteria
- 5.4 Recruitment and informed consent
  - 5.4.1 Script and study information provided
- 5.5 Randomization and treatment allocation
- 5.6 Placebo intervention
- 5.7 Post-baseline meetings with study team
- 5.8 Blinding
- 5.9 Urine toxicology screens

6.0 OUTCOME ASSESSMENTS
- 6.1 Primary outcome
- 6.2 Secondary outcomes
  - 6.2.1 Treatment retention
  - 6.2.2 Self-reported drug use
  - 6.2.3 Subjective Opiate Withdrawal (SOWS)
  - 6.2.4 Objective Opiate Withdrawal (OOWS)
  - 6.2.5 Craving
  - 6.2.6 World Health Organization Quality of Life Assessment (WHOQOL-BREF)
  - 6.2.7 Sleep quality
- 6.3 Other clinical and exploratory outcome assessments
  - 6.3.1 Adapted Credibility/Expectancy Questionnaire
  - 6.3.2 Baseline and post-baseline drug use history and assessment
  - 6.3.3 Behavioral Inhibition/Activation System Scales (BIS/BAS)
  - 6.3.4 Pain Catastrophizing Scale (PCS)
  - 6.3.5 Cleveland Clinic Constipation Scoring System
  - 6.3.6 Placebo compliance assessment
  - 6.3.7 Methadone Symptom Severity Checklist
  - 6.3.8 Monetary Choice Questionnaire (MCQ)
  - 6.3.9 Barratt Impulsivity Scale, version 11 (BIS-11)
  - 6.3.10 Exit survey

7.0 DATA ANALYSIS
7.1 Sample size calculation
7.2 Data analysis plan for pre-specified primary outcome of methadone dose
7.3 Data analysis plan for secondary outcomes
7.3.1 Treatment retention
7.3.2 All other secondary outcomes
7.4 Data Collection: Retention, quality management and storage

8.0 DATA AND SAFETY MONITORING PLAN
8.1 Data collection and quality assurance plan
8.2 Collection and reporting of non-serious adverse events and serious adverse events
8.2.1 Adverse events
8.2.2 Management of serious adverse events or other study risks
8.2.3 Trial stopping rules
8.3 Data Safety Monitoring (DSM)
8.3.1 Quality assurance plan
8.3.2 Responsibility for data and safety monitoring
8.3.3 Data Safety Monitoring Board (DSMB)

9.0 PROTECTION OF HUMAN SUBJECTS
9.1 Behavioral (instrument) data collected
9.2 Electronic Health Record (EHR) data collected
9.3 Intervention
9.4 Materials: Placebo pills
9.5 Risks to human subjects
9.6 Adequacy of protection against risks
9.6.1 Informed Consent
9.6.2 Protections against risk
9.6.3 Specific measures taken to mitigate risk
9.6.3.1 Loss of Confidentiality
9.6.3.2 Breach of privacy
9.6.3.3 Risks associated with psychological questionnaires
9.6.3.4 Risks associated with the time-limited nature of the study
9.6.3.5 Unforeseen adverse reactions to the placebo pills
9.6.3.6 Unknown Risks
9.6.4 Vulnerable Subjects
9.6.5 Inclusion of women and minorities
9.6.6 Inclusion of individuals across the lifespan
9.7 Potential benefits of the proposed research to participants and others
9.8 Importance of the knowledge to be gained

10.0 BIBLIOGRAPHY

11.0 APPENDIX I – Script utilized to describe the conceptual basis of the placebo effect

1.0 BACKGROUND

In 2014, 47,055 drug overdose deaths occurred in the United States, and 61 percent of these deaths were the result of opioid use, including prescription opioids and heroin[1]—a near-quadrupling of opioid-related deaths since 1999. The incredible surge in deaths owing to Opioid Use Disorder (OUD) has caused many federal and state agencies to identify a "heroin epidemic"—a crisis facing our nation so great that it is considered by many to be one of the largest looming threats to public health today. Methadone treatment (MT) is the most highly researched and evidence-based treatment for OUD and has become the mainstay for the treatment of opioid dependence with strong scientific recommendation for continued widespread availability. By binding to μ-opioid receptors, methadone works to alleviate opioid withdrawal. Yet there is great individual variability in MT response. While many patients have positive outcomes with low to moderate doses of methadone[2] (e.g., ~30 mg/day), effectively allowing them to maintain sobriety for years on end, most patients need much higher doses of methadone to maintain their craving and drug-seeking behavior.[3,4] Unresolved medical debates on whether "more is better" provide no clarity on this issue, and with no generally accepted optimal dose prescription, clinicians titrate MT dose to a subjective patient behavioral effect—a practice that often translates to the prescription of very high doses of methadone, affecting both treatment cost and public health policy.

These higher doses of methadone come at an unfortunate cost and are associated with a long list of common side effects and cardiotoxicity. High-dose MT side effects include constipation, sedation, nausea, and sweating, and are often severe enough to drive many patients to quit their MT program. Adams[5] reported that in a study of treatment failures, 27% of the patients on high-dose methadone reported side effects as a primary reason for leaving treatment. In rare cases, severe cardiac effects have been reported, and cases of QT interval prolongation and Torsade de pointes (a cardiac arrhythmia characterized by rapid, irregular QRS complexes)- have been reported in patients taking very high doses of methadone, underscoring the risk associated with high-dose MT. Reports of increasing methadone-related deaths have only fueled this risk awareness, and have led to greater scrutiny of methadone dosing practices.[6,7] Finally, an estimated 30% of MT patients have severe anxiety related to MT detoxification due to fear of withdrawal and relapse[8,9]: concerns that theoretically could be eased by the implementation of treatment paradigms incorporating low-dose MT. Collectively, these various

issues provide a compelling rationale to explore plausible options to increase the therapeutic window of low-dose methadone.

Recent investigations have yielded greater appreciation of the therapeutic potential of the placebo response. Increasingly more research has turned its focus to study ways in which to ethically harness that potential, and two main strategies have dominated these discussions. One stream of research employs principles of Pavlovian conditioning. By pairing placebo pills and clinical contextual cues (conditioned stimuli) with a physiologically active treatment (unconditioned stimuli), researchers have shown that medication dosages can be lowered without decreasing treatment efficacy. For example, Ader and colleagues demonstrated that placebos extend the effects of corticosteroids in psoriasis patients when given in accordance with a partial reinforcement paradigm.[10] The frequency of disease relapse under this partial reinforcement paradigm was lower (26.7%) than in the control group (61.5%), outcomes which were clinically comparable to the reduction in symptoms induced by a full-dose regimen of corticosteroids (22.2%). Similarly, Perlis and colleagues applied a reinforced therapeutic schedule to medically manage chronic insomnia using 10 mg zolpidem pill with 50% active medication and 50% placebos for 12 weeks. The partial reinforcement group showed the same clinical benefit as the groups randomized to 10 mg or 5 mg or intermittent 10 mg nightly dosing.[11] In a separate study, children with Attention Deficit Hyperactivity Disorder (ADHD) showed a therapeutic benefit when placebo pills were paired with a 50%-reduced dose of amphetamine.[12] Thus, pairing a conditioned stimulus with amphetamines, corticosteroids, or zolpidem produced placebo conditioned responses that allowed individuals to be treated effectively with lower doses of the active medication. To date, this associative learning methodology has not been tested on methadone treatment or pain syndromes.

A second strategy to ethically harnessing placebo effects is known as “open-label placebo” administration. In most of these studies, patients in research settings are given placebo pills in an honest and transparent manner, and are told something in accord with, “we know that placebos have powerful effects in double-blind trials, and we want to test whether placebos work even if a patient knows they’re taking placebos.” Several such experiments have yielded positive results on conditions such as irritable bowel syndrome,[13] chronic low back pain,[14] migraine headache,[15] allergic rhinitis[16] and depression.[17] Open-label placebo administration circumvents many of the ethical problems historically associated with traditional (deceptive) placebo use, including disrespect of patient autonomy, threats to a clinician’s integrity, and

potential damage to societal trust in the medical profession.[18] Yet no study has investigated the efficacy of open-label placebo strategies in a methadone maintenance context. The proposal we submit uniquely combines both approaches.

2.0 PROJECT OVERVIEW

2.1 Brief project overview. The broad goal of this proposal is to enhance treatment outcomes for OUD patients enrolled in a daily outpatient MT program. We will combine two well-known protocols for inducing placebo effects. Implementing principles of pharmacological conditioning,[19,20] we will temporally pair placebo pills with the oral methadone solution that is provided to patients at the clinic, to ultimately use placebos as a conditioned stimulus. Additionally, we will apply an open-label paradigm: we will give the participants information concerning the placebo pill in an honest and transparent manner. This unique approach of combining two powerful protocols (pharmacological conditioning and open-label design) capitalizes on methods that have been robustly shown to enhance treatment outcomes in other patient populations.[13] This proposal will allow us to conduct a proof-of-concept study to harness placebo effects with the ultimate scope of enhancing medication-based clinical treatment of Substance Use Disorder and minimizing methadone escalation.

2.2 Specific Aims. SA1: To compare the impact of pharmacologically conditioned open-label placebo to Treatment as Usual (TAU) on ultimate methadone dose achieved at 90 days. SA 2: To assess the effects of conditioned open-label placebo administration on 90-day MT outcomes and patient retention.

2.3 Significance. The findings obtained from these studies will provide crucial pilot data concerning the effectiveness of placebo interventions implemented in the context of substance use disorders, heretofore unexamined. Additionally, the data will contribute importantly to the growing literature on the translational applications of laboratory findings of manipulations involving the placebo effect in clinical practice by exploring a placebo effect in study participants with substance use disorder. To our knowledge, no data such as this exists in the drug addiction literature. If successful, this project will yield pilot data that will allow us to apply for larger funding opportunities using neuroimaging methodologies, with an aim to explore the mechanisms that underlie the placebo phenomenon in individuals with substance use disorders.

3.0 FACILITIES AND RESOURCES

3.1 University of Maryland Addiction Treatment Center (UMATC). The clinical site for the proposed project is the University of Maryland Addiction Treatment Center (UMATC), an urban outpatient (Level-1) drug treatment center based at the University of Maryland in Baltimore, MD. The UMATC is accredited by the Commission on Accreditation of Rehabilitative Facilities, International (CARF) and certified by SAMHSA as an opioid treatment program (OTP). It is a Maryland Department of Health and Mental Hygiene-certified Opioid Treatment Program that provides Level I (outpatient) treatment services. As the first hospital-based methadone program in the state of Maryland (founded in 1972), the UMATC has long embraced a tradition of folding rigorous, empirically validated approaches into comprehensive strategies for the treatment of OUD – a tradition that has served as the philosophical cornerstone for all clinical programing. Additionally, the Division has been an integral collaborator with the Baltimore Buprenorphine Initiative since its inception in 2006. The UMATC has been located at its current site since 2012. The program is centrally located and accessible by public transportation (MTA and Charm City Circulator) and contains ample free parking. The clinic is approximately 15,000 square feet and offers all three FDA-approved forms of MOUD (methadone, buprenorphine and vivitrol) along with individual and group counseling and psychiatric services. The UMATC utilizes an interdisciplinary treatment team which includes: a family medicine/addiction medicine physician, three licensed addiction psychiatrists, one Nurse Practitioner, a full-time psychologist, three registered nurses, five LPNs, two medical attendings, 11 counselors, one social worker, a peer recovery specialist, one clinic director, three administrative staff members and one program manager (who is also a licensed counselor). The UMATC's quality assurance reviews include the quarterly review of staff licensure and credentials as well as monthly Office of the Inspector General Exclusion List reviews.

3.2 UM School of Pharmacy Good Manufacturing Practice Facility (GMPF). Placebo pills used in this program will be produced by the GMPF. The facility has three 500 square-foot research laboratories, one 500 square-foot GLP laboratory, and six GMP pharmaceutical manufacturing suites. It also includes rooms for the receipt and storage of GMP materials. The labs are equipped to perform pre-formulation research, excipient screening, physical characterization of polymorphs, formulation and process development, GMP manufacturing, packaging and labeling. The analytical labs are equipped for methods development, testing and validation. There are also validated stability cabinets to conduct stability studies in accordance with International Council for Harmonization of Technical Requirements for Pharmaceuticals for

Human Use (ICH) guidelines. For this project, placebo pills will be composed of microcrystalline cellulose PH-102, magnesium stearate, and D&C Red 7 Ca Lake (inert chemicals and a food colorant, ingredients commonly contained in placebo pills manufactured by the pharmaceutical industry). Placebo pills are stored in a locked medications cabinet maintained within the nurses' station at the UMATC.

4.0 CLINICAL OUD DIAGNOSIS AND TREATMENT

Methadone is dispensed daily Monday through Saturday with a take-home dose given for Sunday. All patients admitted to the methadone program are assessed by a nurse practitioner and a board-certified addiction medicine physician and are initiated on a standard protocol for treatment of OUD with methadone. Initial methadone doses are individualized with a typical starting dose of 25-30 mg/day that is increased by 5 mg every other day to a targeted holding dose (~80-120 mg). All dose increases are discussed with the treatment team and approved by the OTP's medical director. Patient dosing encounters are logged in the clinic's electronic health record management platform (Methasoft, Netalytics, Greer, NC) and can be accessed for information relevant to treatment retention. Per standard clinic operating procedures, and in line with SAMHSA methadone dosing guidelines,[21] patients missing two or more consecutive days of treatment had their doses reduced (up to 50%) to ensure safe prescribing. Patients who miss 30 consecutive days of dosing (no-call/no-show) are administratively discharged and considered not retained. Urine toxicology is obtained monthly as standard-of-care per state regulations, is recorded in Methasoft, and includes tests for opiates, oxycodone, and fentanyl. In addition to treatment with medications, the clinic provides counseling, psychiatric, basic primary (wound) care, and social work and health home services. Most patients reside within one of five zip codes that immediately surround the clinic address, and present to the clinic either by referral or self-admission. Approximately 4-8 new patients are admitted into the methadone program per week.

5.0 RESEARCH DESIGN AND METHODS

5.1 Overview of study design. We propose to conduct a proof of principle investigation to test the impact of a conditioned, open-label placebo intervention on methadone treatment outcomes. One-hundred twenty new intakes into methadone treatment for OUD will be approached, consented, and randomly assigned to one of two conditions: conditioned open-label placebo (group C-OLP) plus methadone or methadone/Treatment as Usual (TAU). We will follow them

for three months for a total of five in-person meetings (baseline, two weeks, and 1-, 2- and 3-months post-baseline). For the first two weeks, we will implement principles of pharmacological conditioning whereby placebo pills are temporally paired with the oral methadone hydrochloride solution that is provided to patients at the clinic (conditioning phase). Having established an association and contextualized the placebo as part of the therapeutic experience, placebos are then used as a dose extension pill (week 3 up to three months). Additionally, we are applying an open-label paradigm, giving participants information concerning the placebo pill in an honest and transparent manner. Our primary outcome is methadone dose three months after (baseline) entry into treatment; secondary outcomes include several measures of treatment success including comprehensive urine toxicology screens, self-reported drug use, and treatment retention. We are also capitalizing on this unique patient access opportunity to measure several personality and environmental factors associated with OUD, as well as factors associated with placebo response. A complete schedule of all study-related procedures is presented in Table 1.

Table 1. Assessment timeline

		Phase I (Weeks 1 and 2)		Phase II (Weeks 3+)		
		Meeting (Day Number)				
Activity/ Instrument	Time to Complete	1 Day 0	2 Day 14	3 Day 28	4 Day 56	5 Day 84
Verbal assessment of participant interest	1 minute	X				
I/E confirmation	1 minute	X				
Informed Consent (IC)	5 minutes	X				
HIPAA Authorization	2 minutes	X				
IC Evaluation	3 minutes	X				
Study Script	3 minutes	X				
Placebo Effect Video	2 minutes	X				
Pill Information Sheet	1 minute	X	X*	X*	X*	
Day Expectancy Assessment	<1 minute	X			X	X
Past 2-Week Drug Use Assessment	2-3 minutes	X	X	X	X	X

BIS/BAS	8 minutes	X		X		X
Pain Catastrophizing Scale	5 minutes	X		X		X
Pittsburgh Sleep Quality Index	6 minutes	X		X		X
Cleveland Clinic Constipation Scoring	5 minutes	X	X	X	X	X
WHO Quality of Life Assessment	10 minutes	X	X	X	X	X
Craving Assessment	<1 minute	X	X	X	X	X
SOWS	5 minutes	X	X	X	X	X
OOWS	5 minutes	X	X	X	X	X
Baseline Drug Use History	10 minutes	X				
Randomization	1 minute	X				
Order Form*	1 minutes	X	X	X		
Urine Sample	3 minutes	X	X	X	X	X
Payment Logs	3 minutes	X	X	X	X	X
Placebo Compliance*	<1 minute		X	X	X	X
Methadone Side Effects Checklist	5-7 minutes		X	X	X	X
Monetary Choice Ques.	4 minutes		X		X	X
BIS-11	8 minutes		X			
Post-Baseline Drug Use History	5 minutes		X	X	X	X
Exit Interview	4 minutes					X

*Indicates assessments that were administered only to participants in Placebo (C-OLP) Group

5.2 Study population. Study participants will be 120 men and women OUD adults newly admitted to the UM Addiction Treatment Center (UMATC). Over half of the patient population receiving treatment at the UMATC identify as Black or African American (53.1%) and male (55.8%). New patients will be recruited on their first day of treatment in the clinic (Day 0). Subjects of all races and sexes will be included.

5.3 Eligibility criteria. Inclusion criteria include adults (≥18 years old) meeting DSM-V criteria for diagnosis of moderate-to-severe OUD who are new initiates for treatment with methadone

medication. Exclusion criteria include pregnancy (confirmed with urinalysis), transfer into the clinic from another MT program or hospital, and court ordered treatment. These exclusions will be adopted to avoid extenuating factors that could potentially impact clinical treatment determinations or outcomes.

5.4 Recruitment and informed consent. New patients will be recruited on the first day of treatment in the clinic (Day 0). At the end of their initial intake procedures of their first day, patients will be asked if they are interested in hearing information about a study that is testing a novel approach to enhancing methadone treatment, for which they would receive paid compensation. The PI will contact the intake coordinator by 9:00 AM to receive a list of patients who are new intakes to the clinic and who are willing to hear about the study. Those patients who indicate their interest will be approached by the PI (or the study research assistant) later that morning as they are awaiting their methadone dosing in the waiting area of the clinic (normal waiting times vary from between 30 minutes to an hour, providing ample time for a consent and scripted recruitment). At this point, the PI will introduce herself, and will assess interest in study participation. Interested patients will be directed to a small interview room located proximal to the waiting area, and a few steps from the nurses' station. The door will be closed, and the investigator will determine whether the patient meets study eligibility criteria (I/E confirmation), and if so, will obtain informed consent and an authorization for release of Health Insurance Portability and Accountability Act (HIPAA) information for specific data related to OUD treatment. The investigator will then assess comprehension with an Evaluation to Sign Consent form (IC Evaluation). Participants will be asked to provide a suitable method of contact (phone number, e-mail, or consent to be contacted through a flag placed on the person's name in Methasoft, the electronic health record platform used at the UMATC). At the end of the baseline meeting, participants will be given an appointment reminder card with follow-up meeting dates for the 4 return visits. On the day prior to each of these meeting dates, contact will be made with the participant to remind him/her of the next scheduled appointment with the study team.

5.4.1 Script and study information provided. Patients are fully debriefed of all study procedures during the informed consent process. Participants are informed that their participation in the trial will have no effect on ongoing treatment afforded by the clinic, and further, that they have the right to withdraw from the study at any time with no impact on their clinical treatment. During and following consent, the notion is reinforced to the patients that the

research study is "designed to investigate the efficacy of methadone treatment that is enhanced via inner healing processes using placebo effects." A script will be used as a conversational guide to inform patients of the study rationale and procedures (Appendix I). This script has a positive framing and describes in lay terms the science that underlies placebo effects and pharmacological conditioning, with an aim to facilitate the placebo response in a non-deceitful manner. Following the conversational reading of the script, the investigator will ask the participant to view a video of a CBS New York News piece that describes scientific studies of the successful use of the placebo phenomenon as a therapeutic intervention for irritable bowel syndrome. The participant then completes the Day 0 (baseline) assessments. As a final step (and to ensure equal investigator/participant time), prior to treatment allocation, the investigator will give the participant a placebo pill information sheet. This handout will contain explicit instructions on how to take the pill, the pill's formulation, and the contact information for a study team member who will be able to answer any questions that the participant may have about the placebo pill or the study.

5.5 Randomization and treatment allocation. Prior to study inception, random treatment allocation will be generated by an independent investigator in unequal block fashion (60:40 intervention:control). Randomization consists of sequentially numbered opaque envelopes containing treatment assignments drawn from a computer-generated random number sequence. These numbers are used to assign participants to either C-OLP or TAU. Two stacks of envelopes will be created to ensure an even distribution of men and women (N=30/group/sex for a total of 120 random treatment allocations).

Treatment allocation occurs after completion of all Day 0 (Baseline) assessments. The investigator performs allocation by pulling an envelope from the top of the sex-specific stack. Following Day 0 study procedures, and just prior to the first dose of methadone at the treatment window, the investigator conducts a treatment assignment "reveal," opening the envelope and letting the patient know the group to which s/he has been assigned. Group assignment will be revealed with the words: "Great, you're in the placebo group," or "Great, you're in the Control, or Treatment-As-Usual group."

5.6 Placebo intervention. Placebo pills are produced by the University of Maryland School of Pharmacy Good Manufacturing Practice facility. The pills are composed of microcrystalline cellulose PH-102, magnesium stearate, and D&C Red 7 Ca Lake (inert chemicals and a food colorant, ingredients commonly contained in placebo pills manufactured by the pharmaceutical

industry). Placebo pills are stored in a locked medications cabinet maintained within the nurses' station.

Following treatment assignment on Day 0, the investigator fills a placebo pill dispensing form indicating treatment assignment, and the patient is walked to the methadone dosing station. Placebo pills are stored and dispensed by the nursing staff of the UMATC. If the patient is in group C-OLP, the investigator observes the participant taking the placebo pill. In Phase 1 of the study (first two weeks), participants assigned to C-OLP are given one pill, to be taken concomitant with the methadone. In Phase 2 (3 weeks up to 3 months), C-OLP participants continue to take the single (morning/AM) pill and are given a second pill in a bottle as a take-home. They are instructed to take this second pill twelve hours following the first pill, "at home, or wherever they may be." Participants will be asked to return the take-home pill bottle every day for refill. Participants assigned to TAU will also be walked to the methadone window for observed dosing but will not receive a placebo pill. Following dosing, participants will be paid for their participation, and reminded of their next appointment in two weeks.

5.7 Post-baseline meetings with study team. Following the initial (baseline, Day 0) meeting, all participants will be asked to meet with a member of the study team at the following intervals: 14-, 28-, 56-, and 84-days following intake, periods of time that roughly correspond to 2 weeks, 1-, 2- and 3-months of treatment. Participants will be paid $25 for each 30- to 45-minute study visit, for a total possible $125. Because study participants are clients of the UMATC, they must come to the clinic daily for the first several months of their OUD treatment, facilitating follow-up with the study team (the research study team is co-located at the UMATC).

5.8 Blinding. At all stages of the study, methadone dose adjustments are conducted by an independent clinician blinded to treatment allocation and to when a participant is enrolled in the study. Additionally, because treatment allocation occurs only after Day 0 (baseline) assessments are complete, study team members are blind to treatment assignment for all of Day 0 procedures. Finally, data analysts are blind to treatment allocation.

5.9 Urine toxicology screens. Urine drug screening will occur *via* two methods: (1) a point-of-care Quik-tox screen (11 panel; LabCorp) conducted by clinic staff, results of which are reported immediately, conducted at baseline and then at monthly (random) intervals post-baseline; (2) and liquid chromatography–tandem mass spectrometry (LC-MS/MS) testing for a panel of more than 240 drugs, including new psychoactive substances, as well as other illicit and prescription

drugs. All testing is conducted by the Division of Forensic Toxicology, Armed Forces Medical Examiner System and coordinated by the University of Maryland, College Park Center for Substance Abuse Research (CESAR) staff. Urine is collected from participants during each of the five meeting times, and the Quik-tox screen is conducted only on samples from meeting one (baseline); the LC/MS/MS testing is conducted on all samples (baseline and 2 weeks, and 1-, 2- and 3-months post-baseline screening).

6.0 OUTCOME ASSESSMENTS

6.1 Primary outcome. Mean dose of methadone at 3 months (90 days) post-baseline (entry into treatment) will be recorded. These data are obtained from patient charts documenting daily records of methadone dose dispensed.

6.2 Secondary outcome measures. Secondary outcome measures include assessments of treatment retention, self-reported drug use, opioid withdrawal, craving, quality of life, and sleep. All measures will be obtained via facilitated self-report at all five time points, except for sleep, which will be measured only at baseline and one- and three-months post-baseline to maintain fidelity to the instrument's measure of "past-month" sleep.

6.2.1 Treatment Retention. Clinic attendance data will be obtained from participant Methasoft records. Three-month retention will be measured as a binomial variable (retained in treatment at the 90th day, yes/no). For descriptive purposes, retention also will be counted as the total number of days retained in treatment, starting at the participant's intake date, up to 3 months (90 days). The study team adopts the clinic's definition of discharge: 30 continuous days of treatment absence. For participants who are not retained in treatment at the 90th day, the last visit to the clinic prior to their discharge will be logged as the final day in treatment.

6.2.2 Self-reported Drug Use. Drug use is assessed via self-report of past-two-week use of four common substances: opiates (including heroin, fentanyl, and non-prescribed opioid use), cocaine, benzodiazepines, alcohol, and a fifth broad category of "other." The total number of days used (out of a total possible 14) will be recorded.

6.2.3 Subjective Opiate Withdrawal Scale (SOWS). The SOWS is a 16-item self-administered rank assessment of the severity of acute symptoms of opiate withdrawal, with anchor scores of 0 and 4 corresponding respectively to "not at all" and "extremely," for a possible total score of 64.[22]

6.2.4 Objective Opiate Withdrawal Scale (OOWS). The OOWS is a 13-item experimenter-administered assessment of objective symptoms of opiate withdrawal (e.g., yawning, perspiration); symptom presence is scored a "1" for a possible total score of 13.[22]

6.2.5 Craving. Participants will rate their craving for drugs using a one-item craving visual-analog scale adapted from MacKillop and Lisman (2008) and Jobes et al (2015), using the wording: "Please rate how intensely you want to use drugs right now; 0 = "I don't want to use drugs at all," and 100 = "I really want to use drugs." The single-item VAS generates especially robust and reliable results, perhaps on account of its minimal response burden (Jobes et al., 2015) and minimization of test-retest effects.[23,24]

6.2.6 Abbreviated World Health Organization Quality of Life Assessment (WHOQOL-BREF). The WHOQOL-BREF is a 26-item cross-cultural quality of life profile with scores that measure the following broad domains: physical health, psychological health, social relationships, and environment. The WHOQOL-BREF is a shorter version of the original instrument and is better suited for use in large research studies or clinical trials, and has been used previously in the opioid use disorder context and particularly, in this clinic.[25]

6.2.7 Sleep quality. Sleep quality will assessed using the Pittsburgh Sleep Quality Index (PSQI),[26] a 19-item questionnaire to assess past-month sleep quality and disturbances. The 19 self-rated items are combined to form 7 "component" scores, each of which has a range of 0-3 points. In all cases, a score of "0" indicates no difficulty, while a score of "3" indicates severe difficulty. The 7 component scores are then added to a global score, with a range of 0-21 points.

6.3 Other clinical and exploratory outcome assessments

6.3.1 Adapted Credibility/Expectancy Questionnaire. Expectancy will be assessed using a scale asking participants to rate their answers to 3 questions that assess how much they believe that the placebo intervention that they are receiving will help the symptoms that they most want to see improve, as originally implemented by Devilly et al.[27]

6.3.2 Baseline and Post-Baseline Drug Use History and Assessment: a comprehensive assessment of substance use history and treatment, environmental and psychosocial risk factors, and recent use of more than 30 commonly used licit and illicit drugs.

6.3.3 Behavioral Inhibition/Activation System Scales (BIS/BAS)[28]: a 24-item assessment of behavioral inhibition (BIS) and approach (BAS), wherein participants indicate on a four point Likert-like scale the extent to which each of 24 items describes their behavioral style.

6.3.4 Pain Catastrophizing Scale (PCS)[29]: a 13-item assessment of how pain is subjectively experienced, wherein participants indicate on a four point Likert-like scale the extent to which each of the 13 items describe the thoughts and feelings they have when they are experiencing pain.

6.3.5 Cleveland Clinic Constipation Scoring System[30]: This constipation scoring system has 8 questions that allow patients to subjectively report the severity of their symptoms of constipation, a side effect of methadone that is most frequently reported, and which causes much discomfort.

6.3.6 Placebo compliance assessment: Compliance will be assessed using a VAS asking patients to rate how faithful they were in taking the placebo pill, from 0-100 (0=not faithful at all, 100=completely faithful and took the pill daily at the specified time).

6.3.7 Methadone Symptom Severity Checklist[31]: This assessment is designed to have participants rate from 1-5 (with a rating of 5 being most serious) the severity of 38 symptoms of commonly-reported side effects stemming from the use of methadone.

6.3.8 Monetary Choice Questionnaire (MCQ)[32]: A 27-item assessment of delay discounting. For each item, the participant chooses between a smaller, immediate monetary reward and a larger, delayed monetary reward. The protocol is scored by calculating where the respondent's answers place him/her amid reference discounting curves, where placement amid steeper curves indicates higher levels of impulsivity.

6.3.9 Barratt Impulsivity Scale, version 11 (BIS-11)[33]: A 30-item assessment that yields information regarding three facets of trait impulsivity (i.e., attentional impulsivity, motor impulsivity and non-planning impulsivity). Participants indicate on a four-point Likert-like scale the extent to which each of 30 items describes their overall behavior. A scoring algorithm (with some items reverse-scored) yields 6 first-order and 3 second-order factors, as well as an overall score (a total possible score of 120). Higher values on each of the factors indicate higher levels of impulsivity.

6.3.10 Exit Survey: A 7-item quantitative and qualitative assessment of how the placebo pill was experienced by participants and their thoughts about their experience participating in the study.

7.0 DATA ANALYSIS

7.1 Sample size calculation. We anticipate that dose escalations will be recommended at dose evaluation (approximately three weeks following entry into treatment) for approximately 70% of participants in the TAU control group. With 60 participants per group, we will have power of .80 to detect a difference between groups if the corresponding rate in the intervention group is 44% or lower (i.e., a maximum of 26/60 participants), using a Fisher exact test with a two-tailed alpha of .05. This is a medium-to-large effect, equivalent to an odds ratio of 3.03 or a Cohen's d of .61.

7.2 Data analysis plan for pre-specified primary outcome of methadone dose. Descriptive statistics with central tendencies and spread will be used for continuous variables; distributions and percentages will be used for categorical variables. Group differences in mean dose of methadone (in milligrams) at 90 days (primary outcome) will be tested with unpaired t-tests.

7.3 Data analysis plan for secondary outcomes.

7.3.1 Treatment retention. Chi-square analysis will be used to test group differences for the secondary outcome of treatment retention (continuous, up to 90 days).

7.3.2 All other secondary outcomes. Participants' self-reported days of drug use in the last 14 days was logged at each of the 5 total possible study visits. This information will be analyzed separately for opiates, cocaine, benzodiazepine, alcohol, and "other" drug use. For each variable, a mixed-effects longitudinal regression model will be fit to estimate and compare the mean number of days of reported drug use at each study visit by study group. The model follows:

$$\text{Expected}(\text{Numdays}_{ij}) = \beta_0(\text{Baseline}_{ij}) + b_{0i} + \beta_{C\text{-}OLP2}(\text{C-OLP2}_{ij}) + \beta_{C\text{-}OLP3}(\text{C-OLP3}_{ij}) +$$

$$\beta_{C\text{-}OLP4}(\text{C-OLP4}_{ij}) + \beta_{C\text{-}OLP5}(\text{C-OLP5}_{ij}) + \beta_{C\text{-}OLP2}(\text{TAU2}_{ij}) + \beta_{C\text{-}OLP3}(\text{TAU3}_{ij}) + \beta_{C\text{-}OLP4}(\text{TAU4}_{ij}) + \beta_{C\text{-}OLP5}(\text{TAU5}_{ij})$$

where

Numdays_{ij}	=	Number of days of drug use for the ith person at the jth time point
b_{0i}	=	Mean-0 normal random effect for subject i.

Baselineij = 1 if j=1, 0 otherwise.

C-OLP2ij = 1 if the patient is in the C-OLP group and time=2, 0 otherwise

TAU2ij = 1 if the patient is in the TAU group and time=2, 0 otherwise

and

remaining terms defined analogously.

By including a random effect for participant, the means are implicitly adjusted to account for differential dropout based on prior values of these variables. This model is fit using SAS proc mixed. At each time point, the hypothesis will be tested that the mean of the two groups differed. If we find that all the time-point means differed in the same direction, we would also perform a global (4 degrees of freedom) test of a difference between the groups at all four follow-up time points. For each drug analysis we will only include participants who used the drug at least once in the two weeks prior to baseline. All exploratory outcomes (OOWS, SOWS, Craving and WHO-QOL) will be analyzed in the same way as number of days of drug use, using the mixed effects longitudinal regression model.

7.4 Data Collection: Retention, quality management and storage. Participants are given an appointment card that serves as a reminder of the next date that they are to meet with study staff. Additionally, a member of the study team calls participants one day prior to the designated meeting day to remind them of their appointment. Data are collected in an in-person meeting on paper for each instrument. Following the meeting, data are recorded electronically in an ad hoc project created in RedCAP61, a secure data collection and management application hosted at the University of Maryland, Baltimore. Once recorded, data are verified by a secondary independent observer and subsequently locked to prevent changes from being made. Missing data due to missed meetings are coded as incomplete. The resulting database is imported into SPSS and logical consistency checks are conducted and addressed, and missing values designated. Data collected on paper are de-identified with a Study ID number and stored in a locked cabinet in an off-site location. And electronic identifying information will be password-protected on an encrypted, HIPAA-compliant drive, and all study authors will have access to verified, cleaned and de-identified data sets.

8.0 DATA AND SAFETY MONITORING PLAN

The investigator will monitor enrollment numbers, data collection, and data concerns at regular team meetings and as needed (at least monthly) and will periodically review and ensure overall data quality and integrity. In addition, the systems used to process, and store scanned instruments and data are under two layers of password protection and on a secure off-site server. Formal statistical review of the accruing data will be conducted at the 50% mark of enrollment to check for outliers, coding errors and other problems and correct them, under Dr. Wish's direction. This review will be repeated when all data are entered after enrollment is complete.

8.1 Data collection and quality assurance plan. Study staff will be responsible for collecting the data and entering it into the database behind a firewall. Data will be collected on paper for each instrument and is recorded electronically in an *ad hoc* project that created in REDCap, a secure data collection and management application. All source documents will be checked for completeness and accuracy at the end of each day by a secondary independent observer and subsequently locked to prevent changes from being made. Missing data due to missed meetings will be coded as incomplete. Once any data are entered into the database, any changes made to the database records are automatically recorded into an "audit trail" table. Personal health information with a code to the subject's identity will be kept on a password-protected computer behind a firewall. Only investigators for this study will have access to the data. The name with a link to the code will be kept in a locked file drawer with limited access.

8.2 Collection and reporting of non-serious adverse events and serious adverse events

8.2.1 Adverse events: Adverse events associated with placebo pill treatment emergent events will be assessed systematically at each visit. This will provide detailed information on the potential for adverse events occasioned by placebo treatment. All adverse events will be discussed and reported to the IRB. A serious and unexpected adverse experience during the course of the research project, regardless of cause, will be reported to the IRB within 24 hours. UMB policy mandates that initial notification of the event can be made by telephone but must be followed promptly (within 7 days) with a written report, using a "Report of Adverse Experience" form and a copy of the signed consent document. A copy of the "Report of Adverse Experience" will be sent to the UMB Human Research Protections Office through a formal IRB procedure.

8.2.2 Management of serious adverse events or other study risks: Adverse events will be evaluated at each clinic visit. All non-serious and serious adverse events will be discussed with

the PI as well as with study physicians daily and during weekly case management meetings. Serious adverse events will be reported to the IRB and NIDA according to the reporting guidelines described above.

8.2.3 Trial stopping rules: Throughout the trial, if the participation of a subject is deemed unsafe, he or she will be discontinued from the trial. Examples of unsafe conditions include- serious adverse events that are related to the use of the placebo pill, or psychological stress due to the assessments used in the course of the research. Measures have been taken to minimize any risk to our participants. Subjects are free to discontinue from the study and to refuse to participate in any of the procedures.

8.3 Data Safety Monitoring (DSM)

8.3.1 Quality assurance plan: The research team plans to devise a Standard Operating Procedures (SOP) in manual form to minimize errors and data loss for both pen-and-paper and computerized tasks. The SOP manual will also minimize the risks of misassignment and prevent breaches of subject confidentiality. Collected data will be backed up and maintained daily. This will prevent data loss, enhances communication between staff, and ensures full data retrieval for analyses. Security will be maintained by the database and statistical core within Dr. Belcher's Division (DART), which is maintained by the Department of Psychiatry's Information Systems (Psych IS) unit. Personal health information with a code to the subject's identity will be kept on a password-protected computer behind a firewall. Only investigators for this study will have access to the data. The name with a link to the code will be kept in a locked file drawer with limited access. When data are shared with an outside regulatory body or sponsor, such as during reports, activity log, etc., no identifiers will be used, but the data will only contain a study number and clinical information collected during the study.

Internal monitoring of data entry will be conducted by (a) computer programs run on the database to detect values outside the psychometric parameters of the rating scales; (b) direct inspection and rechecking of all safety data by the study coordinator; (c) scheduled monitoring days by all staff, and (d) regular recalibration sessions and recertification of staff. Written reports are generated for these checks, and a tracking system is implemented to prevent systematic errors. Similar procedures are currently being utilized in ongoing studies and meet standards of Good Clinical Practice as set out by the FDA.

8.3.2 Responsibility for data and safety monitoring: The Data Safety Monitoring Board (DSMB) has an important role in the oversight of participants' safety. The main role of the DSMB will be to:

1. Review the research protocol and plans for data and safety monitoring.
2. Evaluate the progress of the interventional trial, including periodic assessments of data quality and timeliness, participant recruitment, accrual and retention, participant risk versus benefit, performance of trial sites, and other factors that can affect study outcome. Monitoring also should consider factors external to the study when interpreting the data, such as scientific or therapeutic developments that may have an impact on the safety of the participants or the ethics of the study.
3. Make recommendations to the IRB and investigators concerning continuation or conclusion of the trial.
4. Protect the confidentiality of the trial data and the results of monitoring.

8.3.3 Data Safety Monitoring Board (DSMB): A DSMB comprised of three independent faculty members from the Department of Psychiatry will be used for this clinical trial. The DSMB is composed of experts who have conducted randomized controlled trials, two of whom have expertise in substance use disorders. The PI will submit a progress report to the DSMB on an annual basis, which will include a reporting of any adverse events, as well as an update on enrollment, raw data reporting, and outcomes and preliminary analysis if available. Within two weeks of receipt of this progress report, the DSMB will meet (or will stage a teleconference meeting) to review the study's progress. The PI will be present during the first part of the meeting, participating only to provide a verbal update of the study's progress, as well as to answer any questions that the DSMB committee members may have. Following this, the PI will be excused (or she will be asked to leave the teleconference), and the DSMB will conduct a closed-door, private meeting to discuss any pertinent issues. All raw data submitted will be stripped of any identifying information, maintaining participants' confidentiality. The DSMB members will be responsible for monitoring the integrity of the research data and safety environment in accordance with established parameters. The DSMB should monitor the data to assess compliance with the protocol including adherence to protocol participation rules for safety. The DSMB also should monitor the quality and completeness of the data being collected, the frequency of missing or erroneous data, and the presence and frequency of outliers. The monitoring of safety data should include review of adverse events (Aes), serious adverse events

(SAEs), and data reflecting general safety such as clinical laboratory data, treatment retention, and reason for dropout when appropriate. Other parameters are as follows: 1) the quality of the consent process will be monitored — this includes ensuring that changes in the consent process are appropriately addressed with the subject and 2) the maintenance of subject confidentiality will be monitored continuously through the protection of verbal and written subject information. Measures put in place for the protection of subjects' confidentiality will be assessed. The DSMB has the right to make independent representation to the regulatory bodies (HRPO) if there has been a failure in reporting by the principal investigator. The DSMB also has the capacity to instruct the principal investigator to pause or terminate pursuance of research if there is a breach in regulatory guidelines or good clinical practices. The DSMB may provide regular independent reports to the HRPO concerning the protection of human subjects in this study. DSMB review meetings will occur on an annual basis.

9.0 PROTECTION OF HUMAN SUBJECTS

9.1 Behavioral (instrument) data collected. All behavioral assessment data will be collected with a facilitated self-report (i.e., a member of the study team will administer and fill in participant responses to the assessments) during each of the 5 meetings with participants. All administered assessments are described in an earlier section and in Table 1.

9.2 Electronic Health Record Data Collected. Consent will be obtained from each participant allowing for select information to be obtained. These data include: clinic attendance, clinic retention/discharge, and clinic urine drug screen test results at intake and monthly thereafter.

9.3 Intervention: Daily Placebo Pill Dispensing for Placebo Arm. Following treatment assignment on Day 0, the investigator will fill a placebo pill dispensing form indicating treatment assignment, and the patient will be walked to the methadone dosing station. Placebo pills will be stored and dispensed by the nursing staff of the UMATC. If the patient is assigned to the Placebo (C-OLP) arm, the investigator will observe the participant taking the placebo pill. In Phase 1 of the study (first two weeks), participants assigned to OLP will be given one pill, to be taken concurrently with the methadone. In Phase 2 (3 weeks up to 3 months), Placebo arm participants will continue to take the single (morning, or AM) pill, and will be given a second pill in a bottle as a take-home. They will be instructed to take this second pill twelve hours following the first pill, "at home, or wherever they may be." Participants will be asked to return the take-home pill bottle every day for refill. Circumstances may occur under which a participant may

need to be withdrawn from the protocol and include not following instructions given by team members.

9.4 Materials: Placebo Pills. The placebo (C-OLP) pill is produced by the University of Maryland School of Pharmacy Good Manufacturing Practice facility. The pills are composed of microcrystalline cellulose PH-102, magnesium stearate, and D&C Red 7 Ca Lake (inert chemicals and a food colorant, ingredients commonly contained in placebo pills manufactured by the pharmaceutical industry). Placebo pills are stored in a locked (Pxyis MedStation) medications cabinet maintained within the nurses' dosing window area.

9.5 Risks to Human Subjects. Risks of participating in research will be clearly communicated to participants in the informed consent form. These include:

- Loss of Confidentiality (highly unlikely, very serious)
- Breach of privacy (highly unlikely, serious)
- Risks associated with psychological questionnaires (highly unlikely, not very serious)
- Risks associated with the time-limited nature of the study (somewhat likely, not serious)
- Unforeseen adverse reactions to the placebo pills (highly unlikely, moderately serious)
- Unknown Risks (highly unlikely, moderately serious)

9.6 Adequacy of Protection Against Risks

9.6.1 Informed Consent. Before participation in any stage of the research study, participants will be required to read and sign an informed consent form. All subjects will consent in writing; the consent form will be dated and countersigned by a staff member. Consent will entail providing participants with information about the study, its rationale, risks, and potential benefits, and the role of the IRB. The consent form will also contain details of who to contact in case of an adverse event, details of the principal investigator, and information on how to contact the Institutional Review Board to register a complaint. Subjects will be informed that they are participating of their own free will and can withdraw their consent at any time during the study. Participants will then be asked to answer questions presented by trained research personnel about critical study details. Participants will be asked what is expected of them by participating, if they fulfill any of the exclusion criteria, if they are fully aware that participation is voluntary and if they have any questions about the study and procedure. Participants will be able to read the consent form in the quiet and locked interview room with no distractions and will be provided with a copy of their signed forms and the PI's contact information to take with them. Another

copy of the form will be kept on file. Authorized research team members will obtain informed consent and will answer any questions the participant may have. At the end of the consent procedure, they will also ask questions to test the participant's knowledge of the study. Participant understanding will be assessed using the HRPO "Evaluation to Sign Consent Form", available at https://www.umaryland.edu/media/umb/oaa/hrp/documents/study-tools-docs/eval_consent.pdf. Participants will be consented for the procedures that will take place during the entire study period; as such, the consent form will cover all treatments and procedures that will occur within that period of the participant's involvement in the investigation. Subjects will be reminded, however, at each of the meetings with the PI that participation is entirely voluntary, and that s/he has the right to withdraw at any time. Consent will include clearly communicated information that participation in the study will not affect an individual's status as a patient at this, or any other methadone treatment clinic.

9.6.2 Protections Against Risk. All research investigators and staff will maintain current training in human subject protections as IRB mandated, and all will be trained with well-specified policies and procedures regarding participant well-being. Questionnaire items and rating scales will be administered by the P.I. or research assistant after obtaining informed consent. All study team members will receive thorough training on study-specific procedures. Further, our ongoing staff training, supervision, and research procedures include pro-active and sensitive monitoring of participant well-being during research encounters (both research intervention and assessments) and responding appropriately to their needs in a wide range of situations, research protocol adherence, and assessing reporting and responding to all adverse events. At all stages of the study, access to trained psychiatrists and medical personnel with experience dealing with this patient population will be available to respond to any unforeseen crisis and to ensure protections against risk.

We take numerous measures to ensure the wellbeing of study participants. All participants will be informed prior to consent that participation is voluntary and that declining will carry no penalties, and importantly, will not affect their treatment status as a patient receiving care at the UMATC. Further, they will be assured that they are free to decline to answer any question(s) they do not wish to answer and to discontinue study participation at any time. If participants feel uncomfortable or fatigued during a data collection assessment or intervention meeting, or seem so to study staff, they will be encouraged to take a break or to stop the activity as they prefer. All study staff will be trained to stop the interview / intervention meeting if a participant becomes

distressed and will have resources (protocol, on-call, resource contacts) immediately available to help the participant obtain whatever level of support or assistance they require, including crisis intervention if needed. All study procedures will be conducted within the context of the Addiction Treatment Center, which is staffed daily by a work force of doctors, a full-time psychiatrist, nurses, a full-time psychologist, and clinical counselors who are specially trained to work with populations of individuals suffering from substance use (and in particular opioid use) disorder. In the unlikely event that a participant shows signs of crisis (heightened anxiety) due to discomfort with any of the assessments, a counselor or doctor on the floor will be engaged to intervene. Additionally, via frequent conversations and regular team meetings, the investigator will monitor study staff observations of participant reactions to all study procedures that may indicate need for an adjustment in protocol. The IRB will be consulted about these as needed. Finally, we plan to apply for a certificate of confidentiality from the NIH to further protect subjects.

9.6.3.1 Loss of Confidentiality (highly unlikely, very serious): This risk is very low, with numerous safeguards for confidentiality. Steps to protect against this risk will include:

- Using only an assigned code number for personally identifiable information (contact information and name) on any records that shows that an individual took part in the study.
- Electronic data will be password protected.
- Paper copies of data will be stored in a locked cabinet.

A federal certificate of confidentiality will add a layer of privacy to protect personally identifying information from being released by any court-ordered request.

Participants will not be given the results of their questionnaires and will be informed that the reason for this is that the data collected are intended for experimental (and not clinical) purposes. Any study data collected for research purposes will not be shared outside of research staff.

9.6.3.2 Breach of privacy (highly unlikely, serious): All study procedures will occur in the privacy of a closed-door interview room located in the methadone clinic, a few hundred feet away from the nurses' methadone dosing station. This room has been set aside to be used for ongoing behavioral treatment studies at the clinic (currently dedicated to Dr. Belcher for research purposes). Patients receiving methadone are called to the methadone counter

individually, so the chance that an on-looker would observe the participant taking a pill prior to his/her methadone dose or taking a placebo pill in the privacy of their home that evening is unlikely. We will minimize the chances of a breach of privacy by interacting with participants in this closed-door, dedicated research room, and every effort will be made to ensure that study participants do not have contact with individuals who are not a part of the study while participating in study procedures.

9.6.3.3 Risks associated with psychological questionnaires (highly unlikely, not very serious): The questionnaires are short, but there is a chance that a participant could become emotionally upset or tired while taking them. Participants will be told that they do not have to answer any questions that make them feel emotionally upset. To minimize tiredness, participants will be given breaks during the study procedure if needed and will be told that they can cease their participation at any time without needing to provide an explanation. However, we do not anticipate that this will be a major risk: study interviews and questionnaires and the behavioral intervention have been used in prior research and elicit minimal distress or discomfort. They are not harmful or unpleasant. Some participants may experience some embarrassment discussing personal information about their drug use history or when participating in the study interviews. The major costs to participants involve the time required to complete the assessments and to participate in the study procedures. Other possible psychological risks can include distress and heightened sensitivity, which can occur as part of the behavioral treatments, clinical interviews, or completing self-reported ratings and questionnaires.

9.6.3.4 Risks associated with the time-limited nature of the study (somewhat likely, not serious): Individuals who are assigned to the C-OLP (intervention) arm will have an opportunity to elect to continue receiving placebo pills beyond the initial three-months of their participation in the study, whereupon they would receive up to one additional week's worth of the placebo pills (14 pills total), and told to take them PRN, if they feel a strong urge to do so. Participants who find that the placebo pills are helpful to their treatment may experience distress when their participation in the study ceases, and they are no longer able to receive placebo pills. To minimize this risk, psychological counseling will be available to participants at the end of their participation in the trial.

9.6.3.5 Unforeseen adverse reactions to the placebo pills (highly unlikely, moderately serious): The placebo pills are formulated as physiologically inert pills. We do not expect the

pills to have direct physiological effects. But in the highly unlikely event that a participant feels the need to access a doctor in a medical emergency because of having taken the placebo pill, the participant will be asked to dial 9-1-1 and to inform a member of the study team as soon as possible.

9.6.3.6 Unknown Risks (highly unlikely, moderately serious): This study may have risks/discomforts that are not yet known. Study staff will update all participants in a timely manner (within 48 hours) if information comes to light that participation in this study could adversely affect a participant's health or welfare. All participants will be directed to contact a member of the study team regarding any problems related to the treatment. The few risks of study participation will be minimized by careful attention to patient confidentiality and discomfort during the research interview.

9.6.4 Vulnerable Subjects. This study does not enroll vulnerable subjects.

9.6.5 Inclusion of women and minorities. No study subject will be excluded based on gender on race/ethnicity. Based upon the known demographics in the University of Maryland Addiction Treatment Center, we expect the study population to be approximately 55% male and 45% female, 55% Black/African American, and 45% White/Caucasian, with 99% identifying as non-Hispanic ethnicity.

9.6.6 Inclusion of individuals across the lifespan. No children under the age of 18 will be included in the proposed study as our sample consists of treatment-seeking adult individuals diagnosed with an opioid use disorder. There is no age limit for inclusion into the study.

9.7 Potential benefits of the proposed research to participants and others. Participants in the experimental condition may have better treatment outcomes than those in standard care, although there is no guarantee of this outcome. Otherwise, there is no direct benefit to the participant.

9.8 Importance of the knowledge to be gained. There are significant benefits to scientific knowledge associated with the proposed research. Methadone has become a mainstay for treatment of opioid use disorder. But as outlined in other sections of this proposal, retention in treatment is a major problem. The knowledge obtained from this project will yield the foundation for future studies of a potentially cost-efficient clinical solution to this problem of methadone treatment retention. The ratio of risks to benefit is reasonable and justified. The risks to subjects in this study are low. We expect that the intervention will be well tolerated. We will minimize the

risks to subjects participating in this study by adhering carefully to our SOP. The potential to improve treatment outcomes in methadone treatment through this study is considerable given the positive findings reported in other arenas of pain and neuropsychiatric disorder research. Considering the potential benefit to society and the minimal risk to participants, the study's risk/benefit ratio is well within an acceptable range.

10.0 BIBLIOGRAPHY

1. Rudd RA, Seth P, David F, Scholl L. Increases in Drug and Opioid-Involved Overdose Deaths — United States, 2010–2015. MMWR Morb Mortal Wkly Rep [Internet]. 2016 [cited 2019 Mar 11];65. Available from: https://www.cdc.gov/mmwr/volumes/65/wr/mm655051e1.htm

2. Trafton JA, Minkel J, Humphreys K. Determining Effective Methadone Doses for Individual Opioid-Dependent Patients. PLOS Medicine. 2006 Feb 7;3(3):e80.

3. Strain EC, Stitzer ML, Liebson IA, Bigelow GE. Methadone dose and treatment outcome. Drug and Alcohol Dependence. 1993 Sep 1;33(2):105–117.

4. Maxwell S, Shinderman M. Optimizing response to methadone maintenance treatment: use of higher-dose methadone. Journal Of Psychoactive Drugs. 1999 Jun 4;31(2):95–102.

5. Adams RG, Capel WC, Bloom WA, Stewart GT. Heroin addicts on methadone replacement: a study of dropouts. The International Journal Of The Addictions. 1971 Jun;6(2):269–277.

6. Chou R, Cruciani RA, Fiellin DA, Compton P, Farrar JT, Haigney MC, Inturrisi C, Knight JR, Otis-Green S, Marcus SM, Mehta D, Meyer MC, Portenoy R, Savage S, Strain E, Walsh S, Zeltzer L. Methadone Safety: A Clinical Practice Guideline From the American Pain Society and College on Problems of Drug Dependence, in Collaboration With the Heart Rhythm Society. The Journal of Pain. 2014 Apr 1;15(4):321–337.

7. Weimer MB, Chou R. Research Gaps on Methadone Harms and Comparative Harms: Findings From a Review of the Evidence for an American Pain Society and College on Problems of Drug Dependence Clinical Practice Guideline. The Journal of Pain. 2014 Apr 1;15(4):366–376.

8. Milby JB, Gurwitch RH, Wiebe DJ, Ling W, McLellan AT, Woody GE. Prevalence and diagnostic reliability of methadone maintenance detoxification fear. The American Journal of Psychiatry. 1986;143(6):739–743.

9. Eklund C, Hiltunen AJ, Melin L, Borg S. Abstinence fear in methadone maintenance withdrawal: a possible obstacle for getting off methadone. Substance Use & Misuse. 1997 May;32(6):779–792.

10. Ader R, Mercurio MG, Walton J, James D, Davis M, Ojha V, Kimball AB, Fiorentino D. Conditioned Pharmacotherapeutic Effects: A Preliminary Study. Psychosom Med. 2010 Feb;72(2):192–197. PMCID: PMC2850283

11. Perlis M, Grandner M, Zee J, Bremer E, Whinnery J, Barilla H, Andalia P, Gehrman P, Morales K, Thase M, Bootzin R, Ader R. Durability of treatment response to zolpidem with three different maintenance regimens: a preliminary study. Sleep Medicine. 2015 Sep 1;16(9):1160–1168. PMCID: PMC4709332

12. Sandler AD, Bodfish JW. Open-label use of placebos in the treatment of ADHD: a pilot study. Child Care Health Dev. 2008 Jan;34(1):104–110. PMID: 18171451

13. Kaptchuk TJ, Friedlander E, Kelley JM, Sanchez MN, Kokkotou E, Singer JP, Kowalczykowski M, Miller FG, Kirsch I, Lembo AJ. Placebos without deception: a randomized controlled trial in irritable bowel syndrome. PLoS ONE. 2010 Dec 22;5(12):e15591. PMCID: PMC3008733

14. Carvalho C, Caetano JM, Cunha L, Rebouta P, Kaptchuk TJ, Kirsch I. Open-label placebo treatment in chronic low back pain: a randomized controlled trial. Pain. 2016;157(12):2766–2772. PMCID: PMC5113234

15. Kam-Hansen S, Jakubowski M, Kelley JM, Kirsch I, Hoaglin DC, Kaptchuk TJ, Burstein R. Altered placebo and drug labeling changes the outcome of episodic migraine attacks. Sci Transl Med. 2014 Jan 8;6(218):218ra5. PMCID: PMC4005597

16. Schaefer M, Harke R, Denke C. Open-Label Placebos Improve Symptoms in Allergic Rhinitis: A Randomized Controlled Trial. Psychother Psychosom. 2016;85(6):373–374. PMID: 27744433

17. Kelley JM, Kaptchuk TJ, Cusin C, Lipkin S, Fava M. Open-label placebo for major depressive disorder: a pilot randomized controlled trial. Psychother Psychosom. 2012;81(5):312–314. PMCID: PMC3813004

18. Blease C, Colloca L, Kaptchuk TJ. Are open-Label Placebos Ethical? Informed Consent and Ethical Equivocations. Bioethics. 2016 Jul;30(6):407–414. PMCID: PMC4893896

19. Colagiuri B, Schenk LA, Kessler MD, Dorsey SG, Colloca L. The placebo effect: From concepts to genes. Neuroscience. 2015 Oct;307:171–190.

20. Colloca L, Enck P, DeGrazia D. Relieving pain using dose-extending placebos: a scoping review. Pain. 2016;157(8):1590–1598. PMCID: PMC5364523

21. Substance Abuse and Mental Health Services Administration. Federal Guidelines for Opioid Treatment Programs [Internet]. Rockville, MD: Center for Substance Abuse Treatment, Division of Pharmacologic Therapies; 2015. Report No.: HHS Publication No. (SMA) PEP15-FEDGUIDEOTP. Available from: https://store.samhsa.gov/sites/default/files/d7/priv/pep15-fedguideotp.pdf

22. Handelsman L, Cochrane KJ, Aronson MJ, Ness R, Rubinstein KJ, Kanof PD. Two new rating scales for opiate withdrawal. Am J Drug Alcohol Abuse. 1987;13(3):293–308. PMID: 3687892

23. MacKillop J, Lisman SA. Effects of a Context Shift and Multiple Context Extinction on Reactivity to Alcohol Cues. Experimental and clinical psychopharmacology. 2008 Aug;16(4):322. PMID: 18729687

24. Jobes ML, Aharonovich E, Epstein DH, Phillips KA, Reamer D, Anderson M, Preston KL. Effects of Prereactivation Propranolol on Cocaine Craving Elicited by Imagery Script/Cue

Sets in Opioid-dependent Polydrug Users: A Randomized Study. J Addict Med. 2015 Dec;9(6):491–498. PMCID: PMC4630075

25. Mitchell SG, Gryczynski J, Schwartz RP, Myers CP, O'Grady KE, Olsen YK, Jaffe JH. Changes in Quality of Life following Buprenorphine Treatment: Relationship with Treatment Retention and Illicit Opioid Use. J Psychoactive Drugs. 2015 Jun;47(2):149–157. PMCID: PMC4425232

26. Buysse DJ, Reynolds CF, Monk TH, Berman SR, Kupfer DJ. The Pittsburgh Sleep Quality Index: a new instrument for psychiatric practice and research. Psychiatry Res. 1989 May;28(2):193–213. PMID: 2748771

27. Devilly GJ, Borkovec TD. Psychometric properties of the credibility/expectancy questionnaire. J Behav Ther Exp Psychiatry. 2000 Jun;31(2):73–86. PMID: 11132119

28. Carver CS, White TL. Behavioral inhibition, behavioral activation, and affective responses to impending reward and punishment: The BIS/BAS Scales. Journal of Personality and Social Psychology. 1994 Aug;67(2):319–333.

29. Sullivan MJL, Bishop SR, Pivik J. The Pain Catastrophizing Scale: Development and validation. Psychological Assessment. 1995;7(4):524–532.

30. Agachan F, Chen T, Pfeifer J, Reissman P, Wexner SD. A constipation scoring system to simplify evaluation and management of constipated patients: Diseases of the Colon & Rectum. 1996 Jun;39(6):681–685.

31. Longwell B, Kestler RJ, Cox TJ. Side effects in methadone patients: a survey of self-reported complaints. Int J Addict. 1979 May;14(4):485–494. PMID: 478696

32. Kirby KN, Maraković NN. Delay-discounting probabilistic rewards: Rates decrease as amounts increase. Psychon Bull Rev. 1996 Mar;3(1):100–104. PMID: 24214810

33. Patton JH, Stanford MS, Barratt ES. Factor structure of the Barratt impulsiveness scale. J Clin Psychol. 1995 Nov;51(6):768–774. PMID: 8778124

APPENDIX 1- Script utilized to describe the conceptual basis of the placebo effect

The following script is a conversational guide that will be used to inform the participants of the study and is meant to provide consistency (and thus standardization of treatment) for researchers. This script will follow consent.

Have you heard about the placebo effect? The placebo effect is what is used to describe the positive medicinal benefits of a pill that has no drug or medicine in it. Placebo pills have been shown to help people with certain conditions have less pain, or in some cases, have less symptoms of a disorder, ultimately, making them feel better. This is because placebo pills activate regions of the brain that release its own pain relievers. It's been shown in many science studies that placebo pills have strong mind-body self-healing properties, and several scientific reports have been published showing that the placebo effect on pain and other conditions can be as strong as if the people took medication. We think this placebo effect may also work for curbing the craving and pain of withdrawal that you typically experience from not taking heroin/pain pills. Although we think this is the case, it has never been tested: it is equally possible that the placebo may not work. Typically, researchers don't tell patients when they are going to get a placebo pill. But scientists are finding out more and more that placebo effects can still happen even when the patient knows it is a placebo (called open-label placebo), helping to treat conditions like back pain, migraine headache and irritable bowel syndrome (chronic stomach pain). We suspect this might also be true for addiction… so that's the point of this study, to test this out.

Half of the participants in this study will get just methadone, like treatment as usual, and the other half will get methadone with a placebo pill. The brain has its own "pharmacy" that releases chemicals to alleviate pain, e.g., endorphins and cannabinoids. If you are assigned to the placebo group, and you are taking the placebo pill with the methadone, quickly your body will begin to recognize the pills as being associated with the methadone. We think that the brain will then release its own endorphins when you take the pill alone and might work because of the association that has been created between the pill and the methadone.

This phenomenon occurs even without you being consciously aware of the association that is being made between the placebo and the methadone- it happens automatically. Now all this said, a positive attitude probably helps. But you don't have to believe that this will work. If it works, it will happen automatically, without you even being aware of it.
