# Supplementary material for: Effectiveness of Conditioned Open-label Placebo With Methadone in Treatment of Opioid Use Disorder: A Randomized Clinical Trial
Source: JAMA Netw Open. 2023 Apr 12;6(4):e237099. doi: 10.1001/jamanetworkopen.2023.7099 (PMC10099063; doi:10.1001/jamanetworkopen.2023.7099)
Supplement: Supplement 2. — eTable. Observed (Raw) Means of Secondary Outcomes at Each Time Point: Drug Use, Withdrawal, Craving, Quality of Life, and Sleep (TAU, n = 54; C-OLP, n = 77) [file jamanetwopen-e237099-s002.pdf]

Supplementary Online Content

Belcher AM, Cole TO, Massey E, et al. Effectiveness of conditioned open-label placebo with methadone in treatment of opioid use disorder: a randomized clinical trial. *JAMA Netw Open*. 2023;6(4):e237099. doi:10.1001/jamanetworkopen.2023.7099

eTable. Observed (Raw) Means of Secondary Outcomes at Each Time Point: Drug Use, Withdrawal, Craving, Quality of Life, and Sleep (TAU, n = 54; C-OLP, n = 77)

This supplementary material has been provided by the authors to give readers additional information about their work.

© 2023 Belcher AM et al. *JAMA Network Open.*

eTable. Observed (raw) means of secondary outcomes at each time point: Drug Use, Withdrawal, Craving, Quality of Life, and Sleep ((TAU, n = 54; C-OLP, n = 77).

Domain	Variable	Group	Baseline Mean (SD)	1-Month Mean (SD)	2-Months Mean (SD)	3-Months Mean (SD)
SELF-REPORTED PAST-TWO-WEEK DRUG USE (days)	Opiates	TAU	13.45 (2.27)	5.95 (5.17)	5.03 (5.04)	5.93 (5.71)
		C-OLP	13.29 (2.26)	5.00 (5.08)	4.69 (5.03)	4.42 (5.01)
	Cocaine	TAU	6.94 (6.37)	2.53 (3.84)	3.57 (4.77)	2.28 (3.63)
		C-OLP	4.38 (5.48)	1.93 (3.58)	2.13 (3.68)	2.43 (4.29)
	Benzodiazepines	TAU	1.38 (3.90)	0.61 (2.52)	0.63 (1.96)	0.93 (2.93)
		C-OLP	0.95 (3.34)	0 (0)	0.11 (0.50)	0.15 (0.97)
	Alcohol	TAU	0.94 (2.94)	0.29 (0.84)	0.40 (1.00)	0.17 (0.54)
		C-OLP	1.32 (3.28)	0.63 (2.03)	1.00 (2.56)	0.68 (1.71)
	Other Drug Use	TAU	2.19 (4.58)	1.61 (4.34)	2.30 (5.07)	1.93 (4.57)
		C-OLP	1.79 (4.20)	1.55 (3.79)	1.27 (3.85)	1.49 (4.11)
WITHDRAWAL	Objective Withdrawal	TAU	2.25 (1.76)	1.68 (1.38)	1.70 (1.26)	1.41 (1.24)
		C-OLP	2.38 (1.28)	1.47 (1.23)	1.21 (1.33)	1.09 (1.24)
	Subjective Withdrawal	TAU	31.27 (16.91)	18.13 (17.58)	19.50 (16.97)	16.38 (18.11)
		C-OLP	27.31 (16.74)	13.55 (12.84)	10.54 (11.97)	8.87 (10.64)
CRAVING	Adapted craving score	TAU	13.42 (7.20)	9.37 (7.37)	9.27 (7.92)	8.59 (7.65)
		C-OLP	13.62 (6.66)	6.90 (5.97)	6.56 (6.32)	6.32 (6.08)
QUALITY OF LIFE: WHOQOL-BREF	Physiological Health Score	TAU	47.56 (16.79)	51.69 (14.99)	49.64 (12.49)	52.96 (15.60)
		C-OLP	52.73 (14.45)	53.85 (11.75)	55.17 (10.96)	55.93 (15.22)
	Psychological Health Score	TAU	53.07 (17.03)	61.07 (20.51)	58.06 (17.09)	60.06 (19.40)
		C-OLP	60.23 (15.32)	62.85 (15.50)	61.31 (16.60)	63.44 (14.15)
	Relationships Score	TAU	55.27 (24.95)	56.58 (29.20)	61.11 (27.10)	64.08 (25.89)
		C-OLP	63.20 (24.83)	67.36 (22.77)	68.90 (23.27)	67.92 (22.43)
	Environment Score	TAU	48.58 (21.97)	56.25 (20.34)	54.69 (21.24)	58.08 (19.65)
		C-OLP	58.52 (18.75)	60.89 (18.27)	61.55 (18.25)	62.50 (19.89)
SLEEP	Sleep: Global PSQI Score	TAU	9.90 (4.35)	9.61 (3.72)	n/a	10.10 (4.10)
		C-OLP	10.73 (4.01)	9.53 (3.26)	n/a	8.62 (3.71)

© 2023 Belcher AM et al. *JAMA Network Open.*
