# Supplementary material for: Effectiveness of Conditioned Open-label Placebo With Methadone in Treatment of Opioid Use Disorder: A Randomized Clinical Trial
Source: JAMA Netw Open. 2023 Apr 12;6(4):e237099. doi: 10.1001/jamanetworkopen.2023.7099 (PMC10099063; doi:10.1001/jamanetworkopen.2023.7099)
Supplement: Supplement 3. — Data Sharing Statement [file jamanetwopen-e237099-s003.pdf]

Data Sharing Statement

Belcher. Effectiveness of Conditioned Open-label Placebo With Methadone in Treatment of Opioid Use Disorder. *JAMA Netw Open*. Published April 12, 2023.
doi:10.1001/jamanetworkopen.2023.7099

Data
Data available: Yes
Data types: Deidentified participant data, Other (please specify)
Additional Information: Informed consent form
How to access data: Researchers wishing to access the data or an electronic copy of the informed consent form may submit a statement detailing the proposed use of the data to the lead author: abelcher@som.umaryland.edu
When available: With publication

Supporting Documents
Document types: Informed consent form
How to access documents: Researchers wishing to access the data or an electronic copy of the informed consent form may submit a statement detailing the proposed use of the data to the lead author: abelcher@som.umaryland.edu
When available: With publication

Additional Information
Who can access the data: Researchers whose proposed use of the data has been approved.
Types of analyses: For purposes that are specified in an approved proposal.
Mechanisms of data availability: After approval of a proposal.
